# The Meningococcal Vaccine Candidate Neisserial Surface Protein A (NspA) Binds to Factor H and Enhances Meningococcal Resistance to Complement

**DOI:** 10.1371/journal.ppat.1001027

**Published:** 2010-07-29

**Authors:** Lisa A. Lewis, Jutamas Ngampasutadol, Ruth Wallace, Jane E. A. Reid, Ulrich Vogel, Sanjay Ram

**Affiliations:** 1 Division of Infectious Diseases and Immunology, University of Massachusetts Medical School, Worcester, Massachusetts, United States of America; 2 Trinity College, Dublin, Ireland; 3 Universität Würzburg, Würzburg, Germany; Northwestern University Feinberg School of Medicine, United States of America

## Abstract

Complement forms an important arm of innate immunity against invasive meningococcal infections. Binding of the alternative complement pathway inhibitor factor H (fH) to fH-binding protein (fHbp) is one mechanism meningococci employ to limit complement activation on the bacterial surface. fHbp is a leading vaccine candidate against group B *Neisseria meningitidis*. Novel mechanisms that meningococci employ to bind fH could undermine the efficacy of fHbp-based vaccines. We observed that fHbp deletion mutants of some meningococcal strains showed residual fH binding suggesting the presence of a second receptor for fH. Ligand overlay immunoblotting using membrane fractions from one such strain showed that fH bound to a ∼17 kD protein, identified by MALDI-TOF analysis as Neisserial surface protein A (NspA), a meningococcal vaccine candidate whose function has not been defined. Deleting *nspA*, in the background of *fHbp* deletion mutants, abrogated fH binding and mAbs against NspA blocked fH binding, confirming NspA as a fH binding molecule on intact bacteria. NspA expression levels vary among strains and expression correlated with the level of fH binding; over-expressing NspA enhanced fH binding to bacteria. Progressive truncation of the heptose (Hep) I chain of lipooligosaccharide (LOS), or sialylation of lacto-N-neotetraose LOS both increased fH binding to NspA-expressing meningococci, while expression of capsule reduced fH binding to the strains tested. Similar to fHbp, binding of NspA to fH was human-specific and occurred through fH domains 6–7. Consistent with its ability to bind fH, deleting NspA increased C3 deposition and resulted in increased complement-dependent killing. Collectively, these data identify a key complement evasion mechanism with important implications for ongoing efforts to develop meningococcal vaccines that employ fHbp as one of its components.

## Author Summary

*Neisseria meningitidis* is an important cause of bacterial meningitis and sepsis worldwide. The complement system is a family of proteins that is critical for innate immune defenses against this pathogen. In order to successfully colonize humans and cause disease, the meningococcus must escape killing by the complement system. In this study we show that meningococci can use one of its surface proteins called Neisserial surface protein A (NspA) to bind to a host complement inhibitory protein called factor H (fH). NspA is a protein vaccine candidate against group B meningococcal disease. Binding of fH limits complement activation on the bacterial surface and enhances the ability of the meningococcus to resist complement-dependent killing. Capsular polysaccharide expression decreases fH binding to NspA, while truncation of the core glycan chain of lipooligosaccharide increases fH binding to meningococcal NspA. Loss of NspA results in enhanced complement activation on the bacterial surface and increased complement-dependent killing of meningococci. Our findings have disclosed a novel function for NspA and sheds further light on how this pathogen evades killing by the complement system.

## Introduction

The complement system forms an important arm of innate immune defenses against *Neisseria meningitidis*. The presence of antibody-dependent complement-mediated serum bactericidal activity predicts protection against invasive disease [1]. Individuals deficient in components of the alternative or terminal complement pathways are predisposed to recurrent episodes of meningococcal infection [2], [3], [4], [5]. In order to survive in its human host, the meningococcus must evade killing by complement (either direct lysis by the terminal pathway or complement-dependent opsonophagocytosis).

Capsular polysaccharide expression is probably the most important determinant of meningococcal virulence. Expression of capsular polysaccharide renders the organism more serum resistant [6], [7], although the molecular basis for capsule-mediated serum resistance remains undefined. In addition, scavenging host complement inhibitors by meningococcal membrane proteins constitutes an important mechanism of subverting complement attack. Opc has recently been shown to bind to vitronectin [8] and contribute to serum resistance [9]. Porin (Por) A (PorA) binds to C4b-binding protein, although binding is best observed under hypo-osmolar conditions [10]. The molecule that has received much attention in recent literature is factor H-binding protein (fHbp; also known as LP2086 [11] or Genome-derived Neisserial Antigen (GNA) 1870 [12]) that binds to the alternative pathway inhibitor, factor H (fH) [13], [14]. FH acts as a cofactor in the factor I-mediated cleavage of C3b to the hemolytically inactive molecule iC3b [15], prevents the association of factor B with C3b thereby retarding the formation of the alternative pathway C3 convertase (C3b,Bb) and irreversibly dissociates the alternative pathway C3 convertase once it is formed [16], [17]. Based on its amino acid sequence, fHbp has been classified into 3 variants [12], or into 2 subfamilies [11], or more recently, into seven modular groups [18], [19]. Despite the fairly extensive fHbp sequence variation among strains, representative strains from each variant family bind to fH [13]. The co-crystal structure of variant 1 (subfamily B) fHbp with a fragment of fH revealed an extensive interaction surface of ∼2,860 Å^2^ [14]. fHbp is currently being evaluated as protein vaccine candidate against group B meningococcal disease and has shown promise in Phase III clinical trials [20].

In light of the use of fHbp as a vaccine, it is important to define alternative means of complement evasion that the meningococcus may employ, in particular scavenging fH. fHbp expression levels vary markedly across strains. Additional mechanisms to bind to host fH could undermine the efficacy of fHbp-based vaccines. In this report we have characterized Neisserial surface protein A (NspA) as a second acceptor molecule for fH on meningococci and have established its role in enhancing meningococcal serum resistance. It is noteworthy that NspA has received attention as a possible group B meningococcal vaccine; identification of a novel function for this protein highlights the potential utility of microbial fH binding molecules as vaccine antigens.

## Results

### fH binds to fHbp deletion mutants: evidence for an alternate fH ligand on meningococci

**Figure 1:**
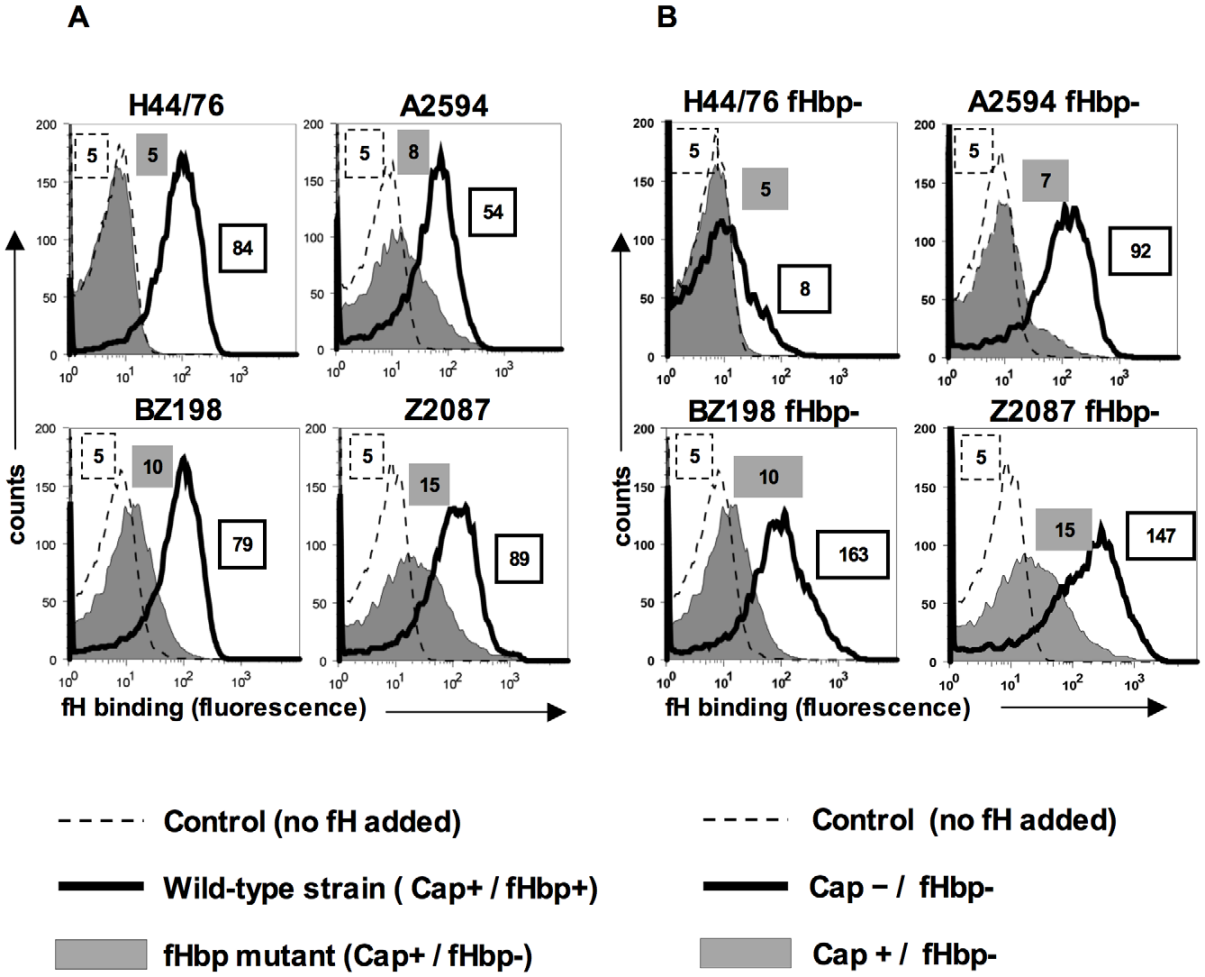
FH binding to fHbp mutants of select *N. meningitidis* strains and their isogenic capsule deficient mutants. **A**. fH binding to wild-type meningococcal strains H44/76, A2594, BZ198 and Z2087 and their fHbp deletion mutants was examined by flow cytometry. Bacteria were incubated with purified human fH at a concentration of 20 µg/ml and bound fH was detected with polyclonal sheep anti-human fH. Representative controls with the wild-type strains where fH was omitted from the reaction mixture are shown by the broken line. The x-axis represents fluorescence on a log_10_ scale and the y axis is the number of events. Median fluorescence is indicated to the right of each histogram. **B**. Capsule expression hinders fH binding to fHbp deletion meningococcal mutants. *fHbp* deletion mutants of encapsulated strains H44/76, A2594, BZ198 and Z2087 and their isogenic unencapsulated mutants were incubated with fH at a concentration of 20 µg/ml and bacteria-bound fH was detected with sheep anti-human fH. fH binding to the encapsulated (Cap+) strains is shown by the shaded histogram and binding to the isogenic unencapsulated (Cap−) mutant is shown by the solid line. Representative controls with the wild-type strains where fH was omitted from the reaction mixture are shown by the broken line. The x-axis represents fluorescence on a log_10_ scale and the y axis the number of events. Median fluorescence is indicated to the right of each histogram.

Human fH binds to the meningococcal surface molecule, fHbp and meningococcal strains, such as H44/76, do not show any detectable binding of fH by flow cytometry following deletion of fHbp ([13] and Figure 1A). However, we observed a small but reproducible, albeit statistically insignificant, binding of fH to fHbp mutants of some meningococcal strains, such as A2594, BZ198 and Z2087 by flow cytometry (grey shaded histograms, Figure 1A) relative to control histograms (Figure 1A, histograms depicted by broken lines). The amount of fH that bound to the fHbp deletion mutants of these strains was reduced compared to their wild-type fHbp expressing parents (histograms depicted by solid lines, Figure 1A). By contrast, fH binding to the fHbp deletion mutant of strain H44/76 was below the level of detection by FACS. These data indicate that some strains of meningococci may express a second molecule that binds to human fH.

### Expression of capsular polysaccharide decreases binding of fH to fHbp-negative meningococci

Capsule expression in *N. meningitidis* is subject to phase variation [21]. Down-regulation of capsule expression occurs during certain stages of pathogenesis, for example, while traversing the epithelial barrier [22]. Further, constitutively unencapsulated strains are commonly found as carriage isolates [23], [24], [25] and may contribute to the development of naturally acquired immunity. We have previously demonstrated that expression of capsule in group B meningococcal strain H44/76 reduces binding of the complement regulatory binding protein, C4b-binding protein (C4BP) by about 50% [10]. To determine if the expression of capsular polysaccharide similarly affects binding of fH to meningococci that lack fHbp expression, we assessed binding of fH to meningococcal strains in which capsule production had been abrogated. Deleting capsule from the *fHbp* mutants of strains A2594, Z2087, BZ198 and H44/76 revealed that isogenic capsule negative (Cap−) *fHbp* mutants bound more fH than their corresponding capsule expressing (Cap+) counterparts (Figure 1B). Consistent with previous observations [13], [26], deletion of capsule from meningococcal strains that expressed fHbp did not significantly alter binding of fH to meningococci (data not shown). While the fHbp mutants of A2594, Z2087 and BZ198 showed a marked increase in fH binding with loss of capsule, only a minimal increase in fH binding was seen in unencapsulated (Cap−) fHbp mutant of H44/76, suggesting that the second acceptor for fH was expressed in variable amounts across strains. These data show that meningococcal strains possess a molecule distinct from fHbp that serves as a ligand for human fH and that binding of fH to this molecule is inhibited, to some extent, by capsule expression.

### LOS HepI chain length is inversely proportional to fH binding to fHbp-negative meningococci

**Figure 2:**
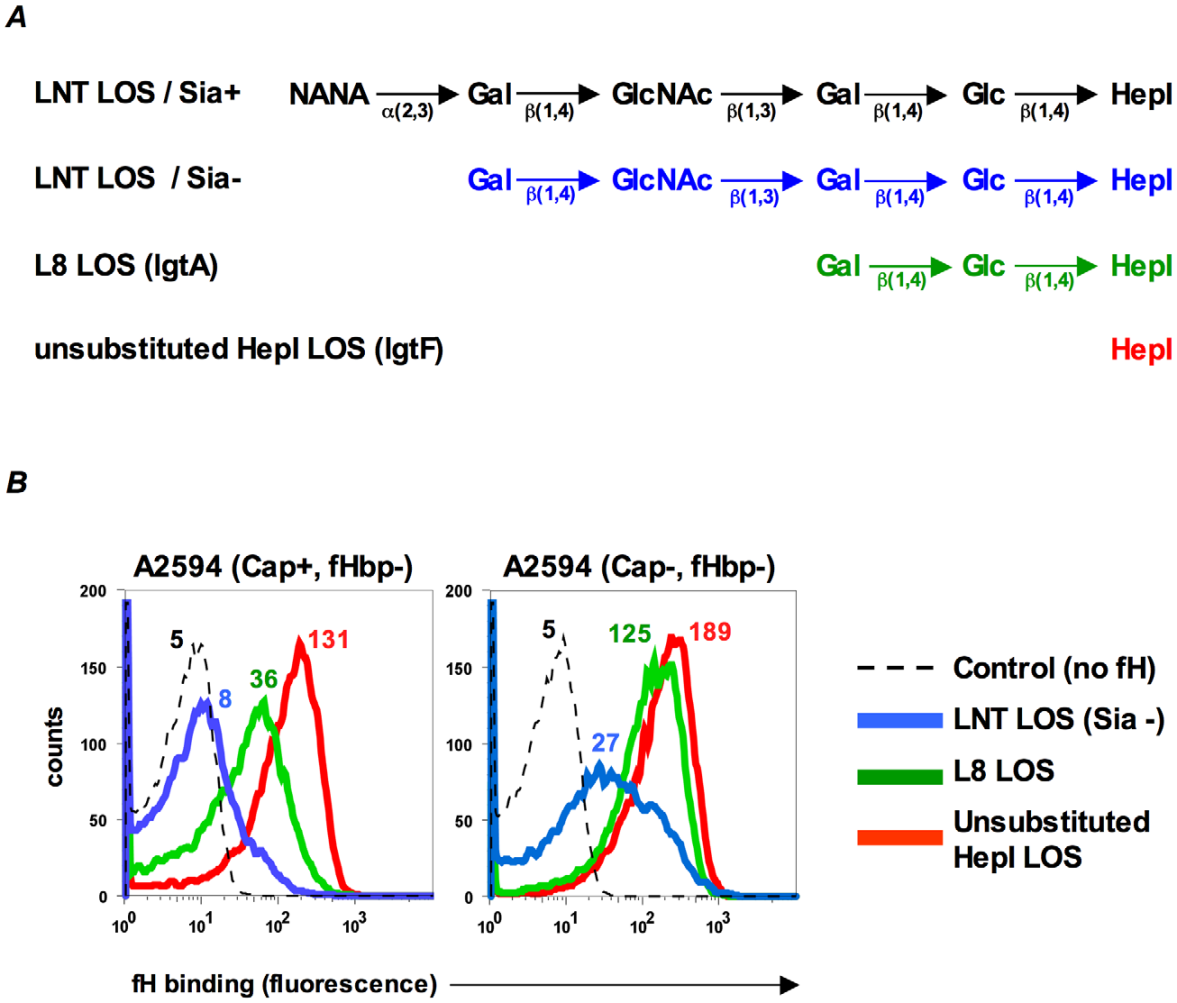
Inverse relationship between fH binding to fHbp mutants and length of HepI glycans extensions. **A**. Schematic depicting the LOS HepI glycan extensions of the strains used in this study. **B**. Binding of fH (10 µg/ml) increases as LOS HepI chain length decreases. fH binding to Cap+ (left panel) and Cap− (right panel) isogenic mutants of A2594 that express either unsialylated LNT LOS (blue graphs), L8 LOS (green graphs) or unsubstituted HepI LOS (red graphs) was measured by flow cytometry. Numbers represent median fluorescence of fH binding from a single representative experiment; color corresponds to the color of the graph. Median fluorescence from three independent experiments was used to perform a Cuzick's nonparametric test for trend across ordered groups. The trend of fH binding increasing as HepI chain length decreases is statistically significant (p-value for trend test p = 0.007). The control (dashed histogram) represents a reaction mixture in which fH was excluded; controls with all mutants yielded similar results and a representative control obtained using the LNT LOS-expressing strain is shown. Axes are as described for Figure 1A.

LPS length can affect binding of complement inhibitors such as fH to bacteria [27]. In Neisseria, many of the genes involved in synthesis of lipooligosaccharide (LOS) are subject to reversible phase variation and a consequence is that the length of glycan extensions from HepI varies [28]. Previous work in our laboratory has shown that altering the length of glycan extensions from HepI affects binding of the complement inhibitor, C4BP, to gonococci [29]. To determine if HepI glycan extensions similarly affect binding of fH to its second ligand on meningococci, we studied the effects of truncating the glycan residues from HepI on fH binding to meningococci that lack fHbp. Wildtype strain A2594 expresses a lacto-N-neotetraose (LNT) extension from HepI and the wildtype LOS is not modified with sialic acid (LNT LOS sia-/Figure 2A, blue). Mutants that express a lactose extending from heptose I (*lgtA* mutants/L8 LOS/Figure 2A, green) or no saccharides off HepI (lgtF mutants/unsubstituted HepI LOS/Figure 2A, red) were created in the background of strains A2594 encapsulated (Cap+) and A2594 Cap−. As seen in Figure 2B, truncation of the HepI chain of LOS results in a progressive increase of fH binding to both Cap+ (left panel) and Cap− (right panel) meningococci. For a given LOS phenotype, the Cap+ mutant bound less fH than the corresponding Cap− mutant, confirming the observation above (Figure 1B) that capsule expression negatively impacted fH binding to the second receptor. In both Cap+ and Cap− backgrounds, the trend of increasing fH binding as the length of LOS HepI chain length decreases was statistically significant (Supplementary Table S1; p-value for trend test = 0.007). The inhibitory influence of capsule on fH binding to the second meningococcal fH receptor decreased as HepI LOS chain length decreased. For example, the differences in fH binding between the Cap+ and Cap− isogenic mutants were least apparent when the HepI of LOS was unsubstituted (red graphs in Figure 2B).

### LOS sialylation enhances fH binding to fHbp-negative meningococci

**Figure 3:**
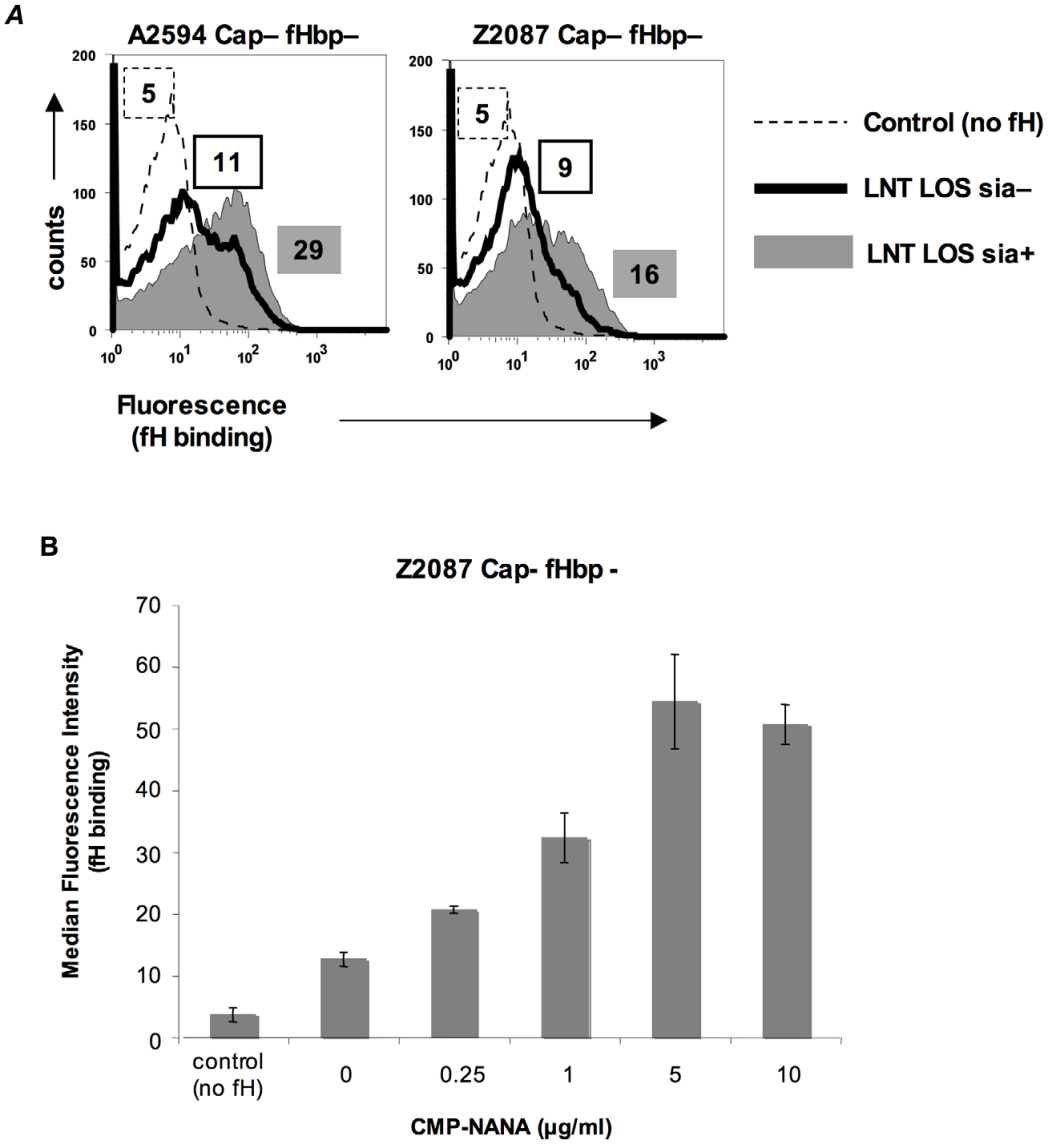
Sialylation of lacto-N-neotetraose (LNT) LOS enhances binding of fH to fHbp-negative meningococci. **A**. fH (10 µg/ml) binding to fHbp deletion mutants of unencapsulated (Cap−) derivatives of strains A2594 (upper panel) and Z2087 (lower panel) grown either with (sia+) or without (sia−) CMP-NANA (5 µg/ml) added to the growth media was measured by flow cytometry using anti-fH mAb 90×. Binding to sialylated (sia+) strains is shown by the shaded graphs and binding to strains without LOS sialic acid (sia−) by the solid line. Controls (no fH added) are shown by the dashed line. Numbers represent the median fluorescence of the corresponding histogram. **B**. fH binding to fHbp− LNT-bearing meningococci is dose dependent and saturatable. Z2087 mynB fHbp (Cap−, fHbp−, LNT LOS) was grown in media containing increasing concentrations of CMP-NANA ranging from 0 to 10 µg/ml. Bacteria were incubated with fH (20 µg/ml) and fH bound to bacteria was detected by flow cytometry using mAb 90×. The average median fluorescence from 3 independent experiments is plotted. Error bars represent standard deviations. The increases in fH binding were statistically significant (p-value<0.05) for all CMP-NANA concentrations prior to saturation. Controls and axes are as described in Figure 3A.

In *N. gonorrhoeae* the modification of LNT LOS with sialic acid dramatically enhances the binding of fH [30], probably by increasing the access of fH to porin [31]. However, LOS sialylation has not been reported to enhance binding of fH to meningococci [26], [31]. Meningococcal strains that belong to groups B, C, W-135 and Y can endogenously sialylate their LNT LOS [32]. Group A strains do not have the capacity to synthesize 5′-cytidinemonophospho-*N*-acetylneuraminic acid (CMP-NANA; the donor molecule for sialic acid) and thus cannot endogenously sialylate their LNT LOS [33], [34]. However, they can scavenge CMP-NANA from the host to sialylate their LNT LOS. To determine if LOS sialylation affects binding of fH to fHbp negative meningococci we analyzed fH binding to fHbp− and Cap− mutants of group A strains A2594 and Z2087. The use of group A strains, that cannot endogenously sialylate their LOS, permitted us to study the effects of increasing amounts of LOS sialylation on fH binding by varying the amount of CMP-NANA added to growth media. As seen in Figure 3A, growth of both strains in CMP-NANA-containing media increased fH binding. Z2087 Cap− fHbp− was then grown in increasing amounts of CMP-NANA (Figure 3B); fH binding increased as CMP-NANA concentrations in the growth media were increased and maximal fH binding was achieved at 5 µg/ml of CMP-NANA. Taken together, the data presented thus far indicate that binding of fH to the putative meningococcal second fH receptor is enhanced by truncation of HepI glycan extensions, sialylation of LNT LOS or loss of capsule expression.

### Identification of NspA as the ligand for fH on fHbp-negative meningococci

**Figure 4:**
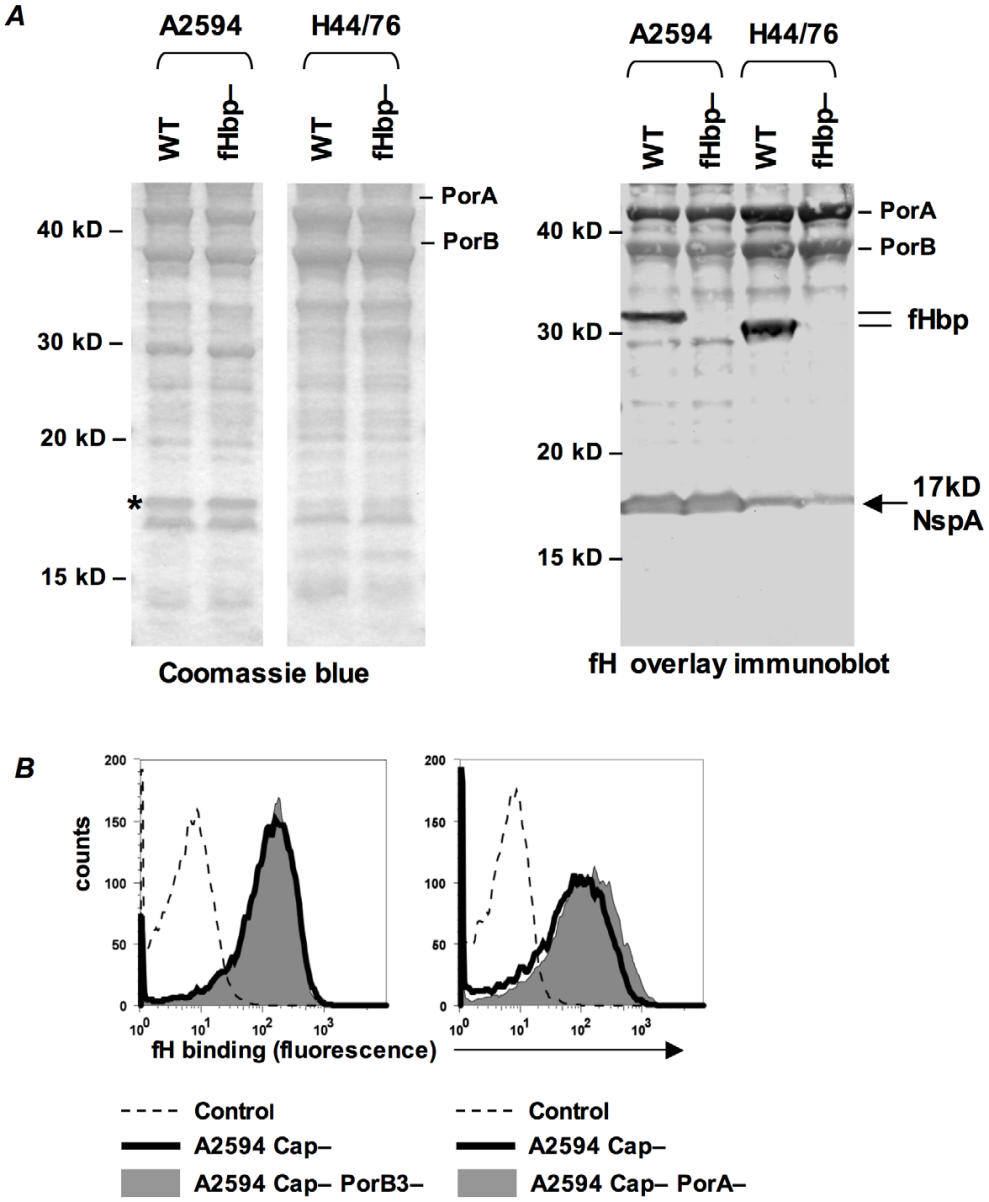
Identification of NspA as a meningococcal ligand for fH. **A**. Membrane extracts from strains A2594 and H44/76 and their fHbp deletion mutants (fHbp−) were separated on a 4–12% Bis-Tris gel, transferred to a PVDF membrane by western blotting and probed with pure human fH (1 µg/ml). Bound fH was detected with sheep polyclonal anti-human fH. The locations of PorA, PorB and fHbp are indicated. A ∼17 kD molecule that also bound fH is indicated by the arrow. This protein was identified by MALDI-TOF analysis of the co-migrating band on a Coomassie blue stained gel (indicated by the asterisk) as NspA (see text). **B**. PorA and PorB are not ligands for fH on intact meningococci. *porA* and *porB3* were deleted from the background of A2594 Cap− (right panel) and A2594 Cap− (left panel), respectively, and fH (10 µg/ml) binding was measured by flow cytometry using anti-fH mAb 90× as the detecting Ab. The shaded graph represents fH binding to the porin deletion mutants and the solid line represents fH binding to the parent strain. Controls and axes are as described in Figure 1A.

Our studies indicate the presence of a second meningococcal receptor for human fH, distinct from the previously described fHbp. A Far Western ligand immuno-blotting assay was used to identify the putative second receptor molecule(s) present on fHbp deletion mutants of *N. meningitidis*. Membrane proteins prepared from strains A2594 (binds fH when its *fHbp* is deleted) and H44/76 (*fHbp* deletion mutant does not bind detectable amounts of fH by FACS) were separated on a 4–12% Bis-Tris gel and transferred to a PVDF membrane. Proteins that bound to fH were identified by probing the membrane with purified human fH and detecting bound fH with an anti-fH Ab (Figure 4A, right). An *fHbp* deletion mutant of each strain was used as a control. We focused on fH-reactive bands that were present in A2594 and A2594 fHbp−, but were either absent or expressed in reduced amounts on H44/76 and H44/76 fHbp−. A prominent fH-binding band of ∼17 kD was apparent in A2594 and A2594 fHbp−. This band was detected, but with lower intensity, in H44/76 and its *fHbp* deletion mutant (Figure 4A, right). This ∼17 kD band was not considered in our previous study where strain H44/76 was employed to identify fHbp as a fH binding molecule [13] because strain H44/76 expresses very low levels of this protein; we focused on the more prominent 29 kD fH-reactive band (fHbp) that was subsequently validated as the fH ligand on intact bacteria. A Coomassie blue stained gel showing the total membrane protein profile of each strain is shown for reference (Figure 4A, left).

To determine the identity of the ∼17 kD fH binding molecule, the region corresponding to the location of the ∼17 kD band was excised from a parallel Coomassie stained gel (indicated by the asterisk, Coomassie blue stained gel, Figure 4A) and this sample was subject to in-gel trypsin digestion and MALDI-TOF analysis followed by peptide mass fingerprinting that was compared with the *Neisseria* proteome. The protein band was defined as Neisserial surface protein A (NspA) using the Peptide Mass Fingerprint program for MS data and the MS/MS Ion Search program for CID data. The peptide ions covered 43% of the total protein sequence and no other statistically significant matches were identified. The data suggest that NspA could bind to human fH.

One caveat of a Far Western assay is that proteins presented in non-native conformations may interact in artificial ways with the ligand, in this case fH, and lead to the detection of “false positive” interactions. The data presented below indicate that NspA is likely the only additional fH ligand present in these strains and additional fH reactive bands present on the Far Western blot (Figure 4A, right) were not analyzed by MALDI-TOF.

Consistent with our previous observations [13], PorA and PorB also bound to fH on the western blot (Figure 4A, right). Purified H44/76 PorB3 binds to human fH by ELISA [35], but neither PorB3 nor PorA bind to fH in the context of intact H44/76 bacteria [13], [26], and we therefore did not anticipate these meningococcal porins to serve as ligands for fH on whole bacteria. The putative surface exposed loops of PorA and PorB show considerable variation across strains [36], [37], [38], [39] and thus it remained possible that the porin molecule(s) of A2594, but not H44/76, served as a ligand for fH. Deleting PorA or PorB3 from the background of A2594 Cap− did not diminish fH binding compared to the respective isogenic Por sufficient parent strains (right and left graphs of Figure 4B, respectively). This suggests that neither Por molecule contributed to fH binding to intact A2594 organisms and that the interaction of these proteins with fH in the Far Western assay is a “false positive”.

NspA shares structural similarities with the Neisserial opacity proteins (Opa) and we sought to determine if Opa might also bind fH. fH binding to an unencapsulated Z2087 strain that expressed Opa and its isogenic Opa negative mutant was indistinguishable (Supplementary data Figure S1), indicating that the Opa proteins were not ligands for fH.

### NspA is a ligand for fH on intact meningococci

Several lines of evidence were used to independently verify that NspA is a ligand for fH on live, intact meningococci.

#### i) Deleting NspA abrogates fH binding to intact fHbp-negative bacteria

**Figure 5:**
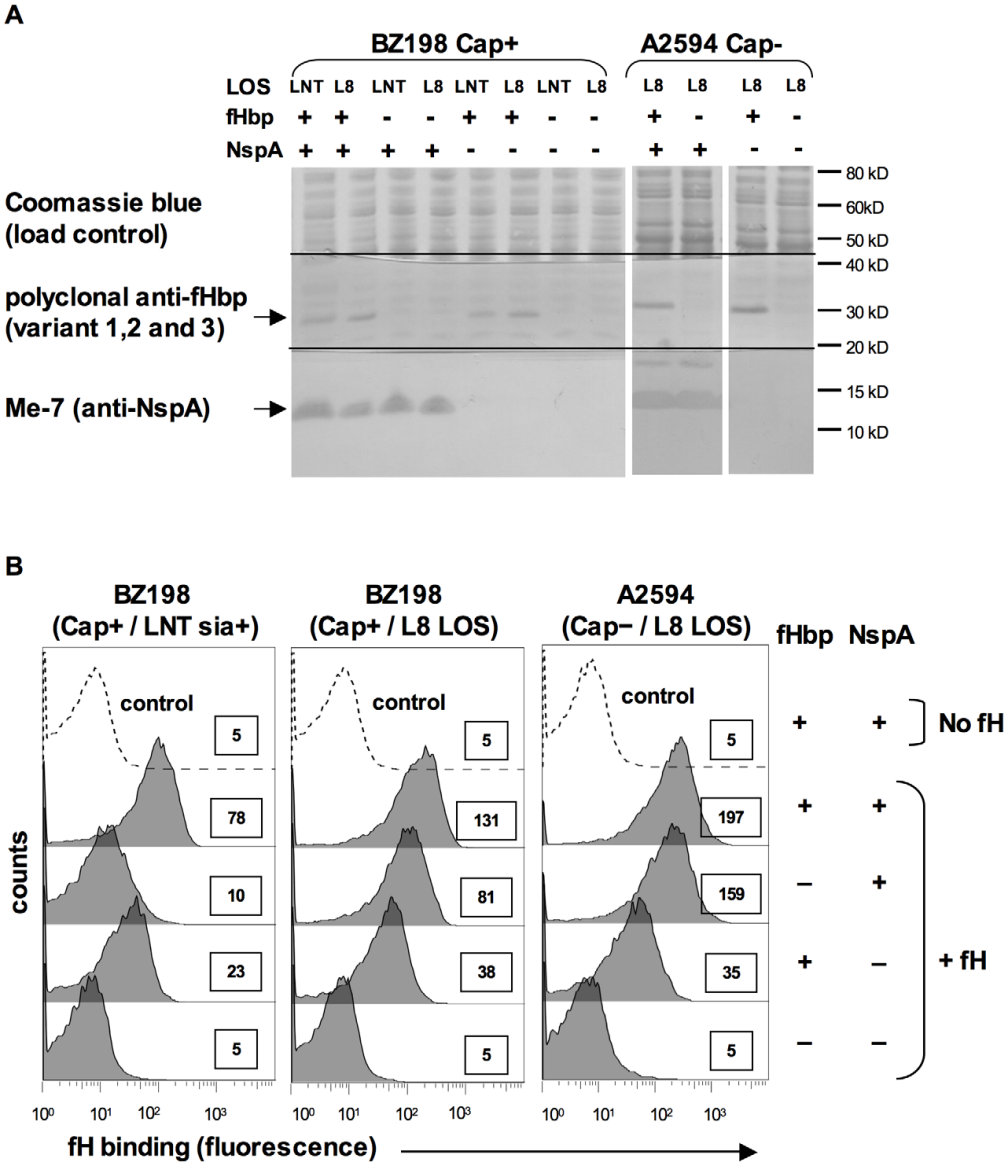
Deleting NspA decreases binding of fH to *N. meningitidis*. **A**. Expression of fHbp and NspA in BZ198 and A2594 derivatives as determined by western blotting of whole cell lysates followed by detection with polyclonal anti-fHbp (variant 1,2 and 3) or anti-NspA mAb Me-7 as indicated. After transfer, proteins migrating above ∼50 kD were stained with Coomassie blue and served as a loading control, proteins migrating between ∼20 kD and 40 kD were probed to detect fHbp and proteins migrating below 20 kD were probed to detect NspA. NspA migrates with an apparent molecular mass of approximately 15 kD when 4–12% Bis-Tris gels are used with MES running buffer. Also, of note, NspA is a heat-modifiable protein and the second larger anti-NspA-reactive band seen in some lanes is the result if incomplete heat denaturation. **B**. Strains BZ198 Cap+/LNT sia+, BZ198 Cap+/L8 LOS and A2594 Cap−/L8 LOS, and their *fHbp*, *nspA* or *fHbp nspA* double mutants were examined for their ability to bind to fH (20 µg/ml) by flow cytometry. The boxed numbers accompanying each histogram represents the median fluorescence of fH binding of the entire bacterial population. Controls (shown by the broken lines) represent fluorescence where fH was omitted from the reaction mixture; all strains yielded similar background binding and, for simplicity, only tracings obtained with the parent strains have been shown. Axes are as described for Figure 1A.

To determine the role of NspA in binding to human fH on intact fHbp-negative meningococci we constructed *nspA* deletion mutations in strains BZ198 and A2594. The BZ198 mutants were constructed in a Cap+ strain that expressed either sialylated LNT LOS (BZ198 is able to endogenously sialylate its LOS) or L8 LOS. The A2594 mutants were constructed in a Cap− strain expressing L8 LOS, to maximize binding of fH. All mutations were constructed in both fHbp+ and fHbp− backgrounds to compare the relative contribution of each of these ligands to fH binding. The expression of fHbp and NspA was verified in all strains by western blot using polyclonal anti-variant 1,2 and 3 fHbp antiserum and anti-NspA mAb Me-7, respectively (Figure 5A). Binding of fH to the *nspA* deletion mutants of BZ198 and A2594 was measured by flow cytometry and, as seen in Figure 5B, deleting *nspA* uniformly decreased fH binding to all strains. No residual fH binding to any of the strains was detected when both fHbp and NspA were deleted. These data, taken together with the direct binding of fH to NspA observed in the Far Western assay, confirmed that NspA served as a ligand for fH on intact bacteria. In addition, these data suggest that the increased fH binding observed in strains that express truncated (L8) LOS also occurred to NspA (i.e., no additional fH ligands were exposed).

LNT LOS sialylation enhanced binding of fH to fHbp-negative meningococci (Figure 3). To determine if the enhanced fH binding observed in sialylated fHbp-negative meningococci was dependent on NspA expression, we examined fH binding to an *fHbp nspA* double mutant. A2594 Cap− fHbp− NspA− with LNT LOS was grown in media containing CMP-NANA and, despite LNT LOS sialylation, there was no increase in fH binding noted by flow cytometry (data not shown). This upholds that the increased fH binding seen when the LNT LOS of the group A fHbp deletion mutants was sialylated (Figure 3A) was not because of fH binding to LOS sialic acid directly, but that the enhanced binding was dependent on the concomitant expression of NspA.

#### ii) Recombinant NspA expressed in E. coli binds to fH and binding of fH to NspA can be inhibited by an anti-NspA mAb

**Figure 6:**
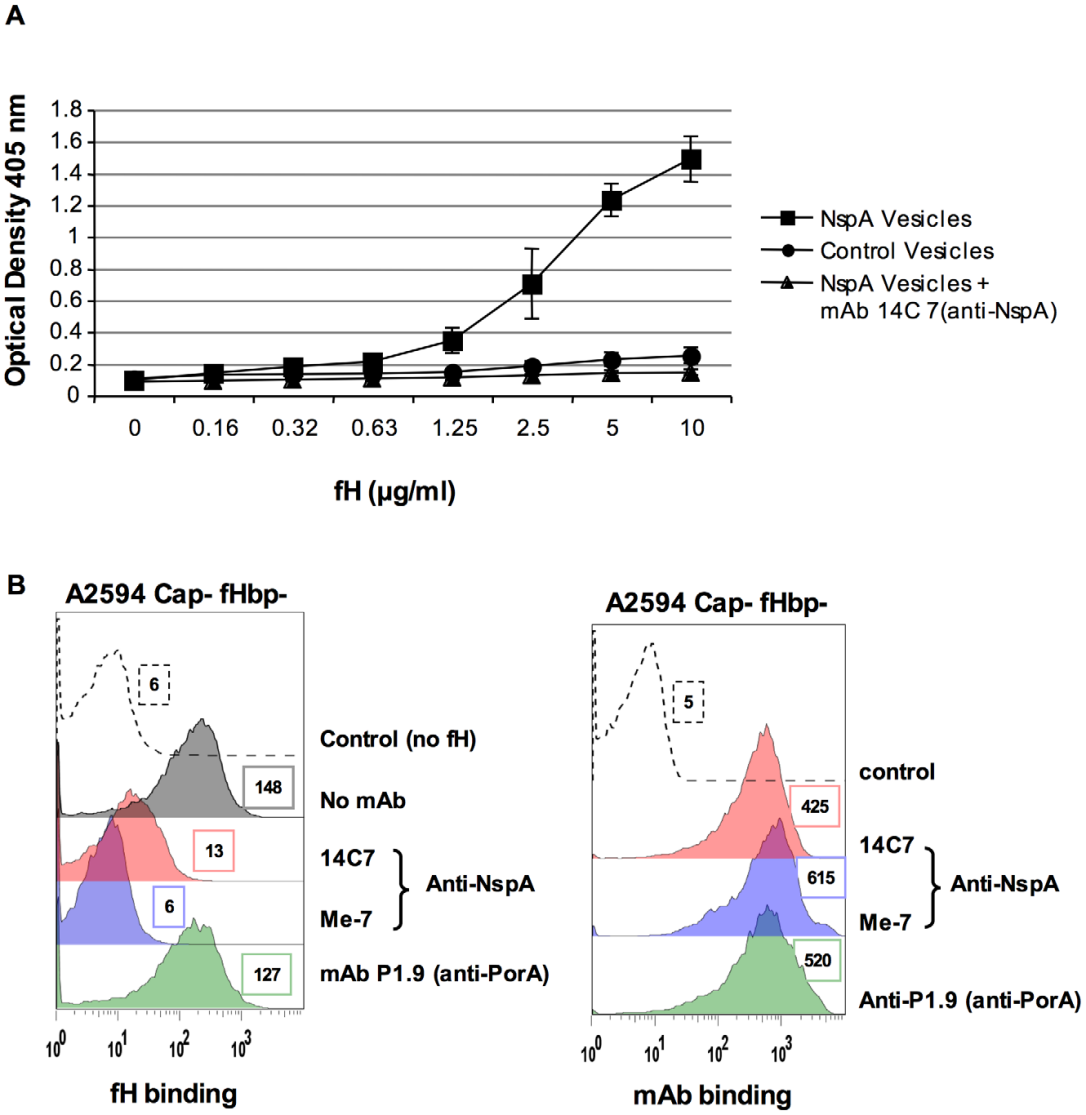
Recombinant NspA expressed in *E. coli* vesicles binds to fH. **A**. Binding of fH to microvesicles prepared from an *E. coli* strain expressing recombinant NspA (squares) or to vesicles prepared from the same *E. coli* strain transformed with the plasmid without the *nspA* gene (circles) was measured by ELISA. Binding of fH to vesicles harboring recombinant NspA was blocked by the anti-NspA mAb 14C7 (triangles). Each data point represents the arithmetic mean of the OD_405nm_ reading from three independent experiments and error bars represent the standard deviation. **B**. Binding of fH to A2594 Cap− fHbp− LNT LOS sia− was measured in the presence of anti-NspA mAb Me-7 (purple shaded histogram), anti-NspA mAb 14C7 (pink shaded histogram) or anti-PorA mAb P1.9 (green shaded histogram); all mAbs were used at a concentration of 30 µg/ml and fH binding was detected using sheep anti-human fH. The control (histogram depicted by a dashed line) represents bacteria incubated with mAb Me-7, followed by addition of anti-fH and anti-sheep IgG FITC. A control where fH binding was measured in the absence of any added mAb is shown by the grey shaded histogram in the left panel. Surface binding of each mAb (shading as described above) to A2594 Cap− fHbp− LNT LOS sia− is also shown. The control (dashed line) represents bacteria incubated with anti-mouse IgG FITC. Median fluorescence is indicated to the right of each histogram.

Microvesicles prepared from an *E. coli* strain expressing recombinant NspA [40] were used to demonstrate direct binding of fH to NspA by ELISA (Figure 6A). Dose-dependent binding of fH to NspA-containing vesicles was observed. In contrast, no binding of fH was observed to vesicles prepared from an *E. coli* strain harboring the plasmid without *nspA* at any of the fH concentrations tested. The specificity of fH binding to NspA containing vesicles was further validated by the ability of the anti-NspA mAb 14C7 to block binding of fH to vesicles containing NspA (Figure 6A).

Additional evidence that NspA was an acceptor molecule for fH on the meningococcal surface was provided by the ability of the anti-NspA mAbs Me-7 and 14C7 to block binding of fH to strain A2594 Cap− fHbp− (Figure 6B). A control mAb (mAb P1.9) directed against the class I outer membrane protein porin A (PorA) did not affect fH binding to bacteria. Similar surface binding of the mAbs used, in the absence of fH, is shown in Figure 6B. Binding of fH in the absence of any added mAb was similar to that seen with mAb P1.9 (shaded grey histogram, left panel, Figure 6B).

#### iii) Increasing NspA expression enhances fH binding

**Figure 7:**
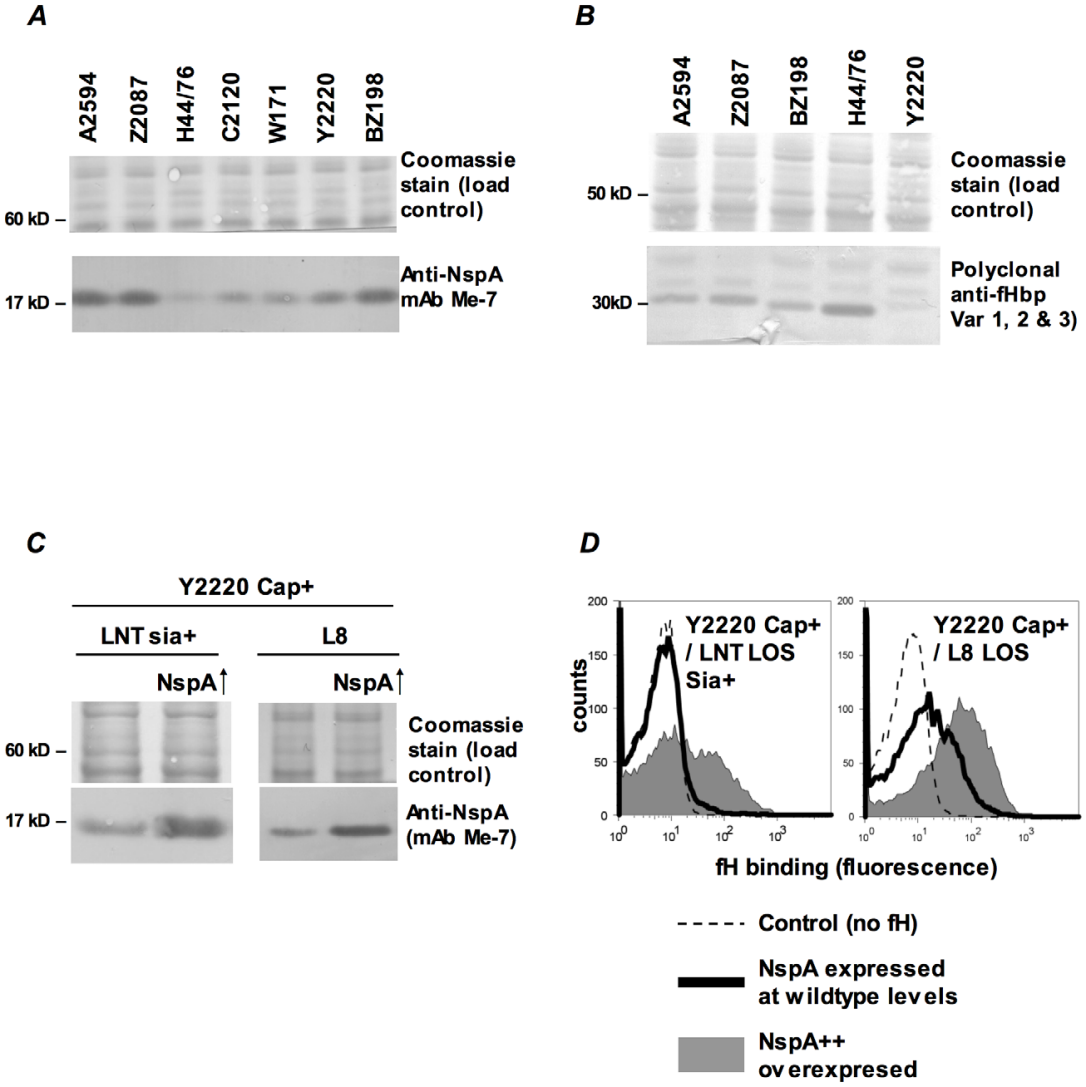
fH binding increases with increasing NspA expression. **A**. Comparison of NspA expression in Cap− derivatives of strains A2594, Z2087, H44/76, C2120, W171, Y2220 and BZ198 by western blotting of whole cell lysates followed by detection with anti-NspA mAb Me-7. After transfer, proteins migrating above 50 kD were stained with Coomassie blue and served as a loading control. **B**. Comparison of fHbp expression in strains A2594, Z2087, H44/76, BZ198 and Y2220 by western blotting of whole cell lysates followed by detection with polyclonal anti-variant 1, 2 and 3 fHbp. After transfer, proteins migrating above 40 kD were stained with Coomassie blue and served as a loading control. **C**. Overexpression of NspA. The *porA* promoter was used to increase NspA expression in the backgrounds of Y2220 Cap+/LNT sia+ and Y2220 Cap+/L8 LOS. Bacterial lysates were subject to western blotting and probed with mAb Me-7. Similar loading of parent and mutant strains was confirmed by Coomassie staining as described in *A*. **D**. Overexpression of NspA enhances fH binding to Y2220 Cap+/LNT sia+ and Y2220 Cap+/L8 LOS. fH binding to the parent strain, expressing wildtype levels of NspA, is shown by the solid line and binding to the NspA overexpressing isogenic mutant is shown by the grey shaded histogram. A representative control (no added fH) is shown by the dashed line. Axes are as described in Figure 1A.

NspA expression levels vary among meningococcal strains [41] and it appeared that the amount of fH binding mirrored expression levels. Consistent with the high levels of fH binding seen to their *fHbp* deletion mutants, strains BZ198, A2594 and Z2087 were also high NspA expressers as seen on western blot analysis of whole bacterial lysates (Figure 7A). Of the strains tested, H44/76 expressed the lowest levels of NspA, while C2120, W171 and Y2220 all expressed intermediate levels of NspA (Figure 7A). The results obtained by western blotting were confirmed by flow cytometry using anti-NspA mAb Me-7 (data not shown). Expression level of fHbp also varies among meningococcal strains [12] and fHbp expression in several strains is shown (Figure 7B). As noted previously, H44/76 is a high fHbp-expressing strain [13], [42] while fHbp expression in Y2220 barely exceeds the level of detection. The expression of fHbp and NspA did not vary within a strain in response to the genetic manipulations described herein (Supplementary Figure S2).[Fig ppat-1001027-g008]

**Figure 8 ppat-1001027-g008:**
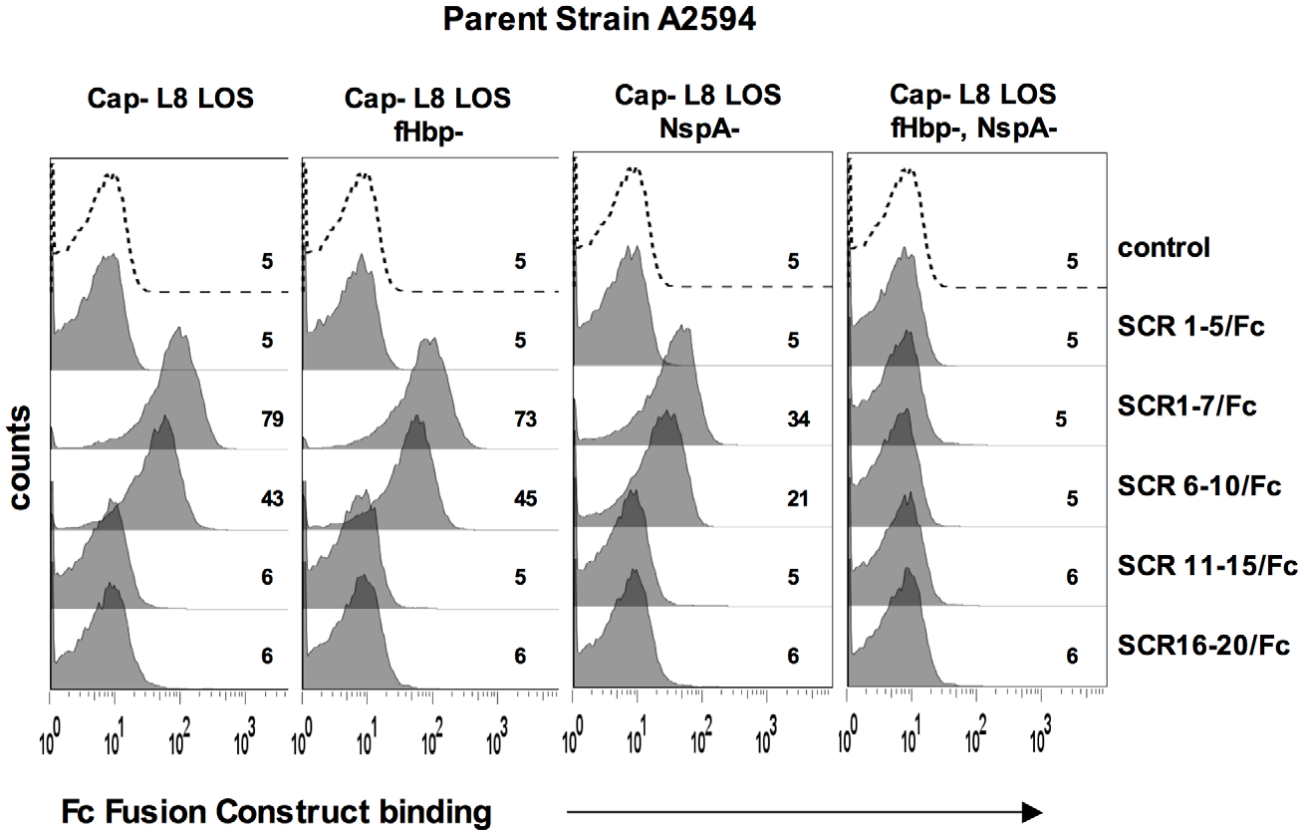
Binding of fH/Fc fusion proteins to *N. meningitidis* fHbp and NspA mutants of A2594. Binding of fH/Fc fusion constructs to A2594 Cap− L8 LOS and to its fHbp−, NspA− and fHbp− NspA− double mutants was assessed by flow cytometry using anti-mouse IgG FITC to disclosure the bound constructs. Strains that expressed either fHbp, NspA or both bound only to fH/Fc fusion constructs that contained SCR 6 and SCR 7 (SCR 1–7/Fc and SCR 6–10/Fc) of fH. In all graphs, the *x*-axis represents fluorescence on a log_10_ scale and the *y*-axis represents the number of events. No fusion protein is present in the control tube. One representative experiment of at least three independent experiments is shown.

We hypothesized that increasing NspA expression would enhance fH binding. NspA expression was upregulated by placing *nspA* under control of the *porA* promoter in the background of wild-type strain Y2220 expressing sialylated LNT LOS (Cap+/LNT LOS sia+) and Y2220 Cap+/L8 LOS (Y2220 *lgtA::kan*). Strain Y2220 was chosen because it expresses very low levels of fHbp [13]. Thus, the effects of NspA over-expression on fH binding could be studied with minimal confounding background fH binding to fHbp. Enhanced NspA expression by the *porA* promoter was confirmed by western blotting (Figure 7C). Increased NspA expression was associated with increased fH binding (Figure 7D). Again, the strain that expressed the shorter (L8) LOS bound more fH that the isogenic mutant with LNT LOS.

Meningococcal strains such as, H44/76, C2120, W171 and Y2220 express low or intermediate levels of NspA, but do not bind fH when fHbp is deleted (data with H44/76 shown in Figure 1A and reference [13], Y2220 shown by solid black histogram in left panel of Figure 7D; data with C2120 Cap+ and W171 Cap+ are not shown). The Cap− mutants of these strains also show minimal fH binding when they express LNT LOS and when *fHbp* is deleted (Supplementary Figure S3). We speculated that further truncation of LOS in these strains would disclose fH binding. In accordance with this hypothesis, truncating LOS (*lgtF* mutants; HepI unsubstituted) resulted in increased fH binding (Supplementary Figure S3).

### NspA mediates binding of fH to meningococci through interactions with fH short consensus repeats (SCRs) 6 and/or 7

FH comprises 20 short consensus repeat (SCR) domains arranged as a single chain [43]. Recently, the cocrystal complex of variant 1 fHbp with fH SCRs 6–7 showed an extensive area of interaction of fHbp with fH SCR 6 and minor points of contact with SCR 7 [14]. Site-directed mutagenesis studies also localized the fHbp binding domain in fH to SCR 6 [44]. To determine the fH SCRs involved in binding to NspA, we utilized fusion proteins that contain contiguous fH SCRs fused at their C-terminus to the Fc portion of IgG2a [44], [45]. The Fc fragment served as a ‘tag’ for symmetric detection of all fusion proteins. The ability of five fH/Fc fusion constructs (SCR 1–5/Fc, SCR 1–7/Fc, SCR 6–10/Fc, SCR 11–15/Fc and SCR 16–20/Fc) to bind to meningococcal strain A2594 Cap− L8 LOS and its isogenic fHbp−, NspA− and fHbp− NspA− double negative mutants, was examined by flow cytometry. Only those fH/Fc proteins that contained SCRs 6 and 7 (SCR 1–7/Fc, and SCR 6–10/Fc) bound to the NspA expressing strains that lacked fHbp. This result indicates that like fHbp, NspA binds to SCR 6 and/or 7. As expected, the SCR 6/7 containing constructs bound to fHbp expressing strains while none of the fH SCR/Fc constructs bound to mutants lacking both fHbp and NspA.

Factor H-like molecule 1 (FHL-1) comprises fH SCRs 1–7 plus four unique additional C-terminal amino acids (SFTL) [46]. FHL-1 also bound to Cap− fHbp− A2594 (Supplementary Figure S4), supporting the conclusion that SCRs 6 and/or 7 play a role in binding of fH to NspA. This finding is consistent with the NspA binding site residing in fH SCRs 6 and/or 7.

Together, these data suggest that SCR 6 and/or SCR 7 are important for binding of fH to NspA. Although less likely, these data do not unequivocally exclude a role for SCRs 8, 9 and 10 in binding of fH to NspA; studies to precisely localize the NspA binding region in fH are underway.

### Species-specificity of fH binding to *N. meningitidis* expressing NspA

**Figure 9:**
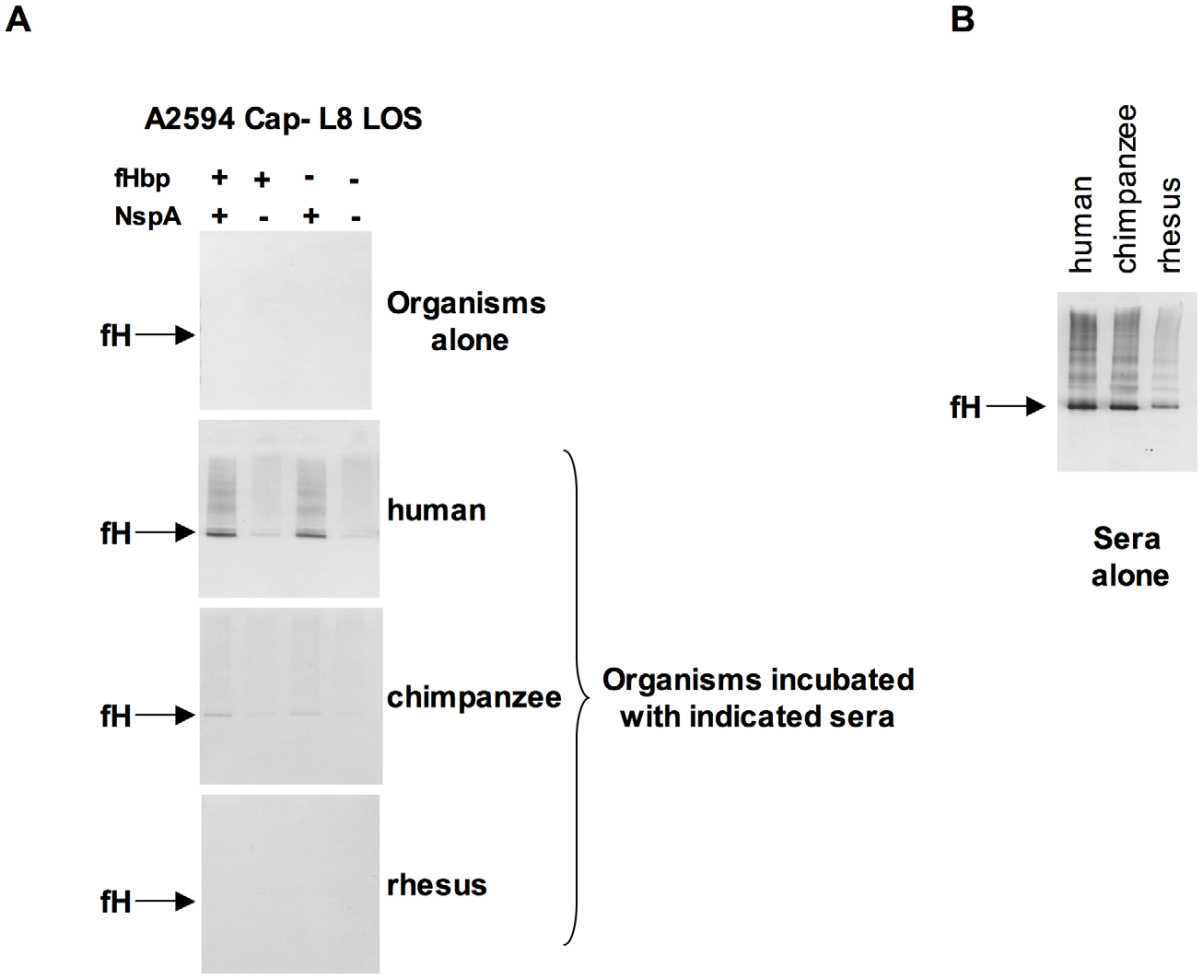
Meningococcal NspA binds selectively to human fH. **A**. Unencapsulated *N. meningitidis* A2594 expressing L8 LOS and its fHbp−, NspA−, and fHbp−NspA− isogenic mutants were incubated with 10% (v/v) heat-inactivated human, chimpanzee and rhesus sera and Western blots were performed using polyclonal goat anti- human fH. **B**. Human, chimpanzee and rhesus serum controls at a dilution of 1/200 (v/v) were performed to ascertain that the goat polyclonal anti-human fH Abs recognized all primate fH tested.

*N. meningitidis* and *N. gonorrhoeae* are exclusively human pathogens and the ability of these pathogens to evade complement-mediated killing in a species-specific fashion may contribute to the narrow host range of infection [45], [47], [48]. We have shown previously that gonococci bind specifically to human C4BP (and in some instances, chimpanzee C4BP) [48] and human fH [45]. Likewise, binding of fH to meningococcal fHbp is specific for human fH [47]. To determine if fH binding to NspA is also species specific we examined binding of fH from different primate species to *N. meningitidis* strain A2594 Cap− L8 LOS and its isogenic fHbp−, NspA− and fHbp− NspA− double-negative mutants by Western blotting (Figure 9A). Strains were incubated with 10% heat-inactivated human or primate sera to assess direct binding of fH to bacteria. Heat inactivation destroys heat labile complement components while leaving fH intact; inactivation of complement is necessary to prevent detection of complement C3b-mediated binding of fH to meningococci. Bound fH was detected by Western blot using polyclonal goat anti- human fH Abs. This Ab reacts with fH in the primate sera tested (Figure 9B) and as previously reported, detection of rhesus fH was slightly weaker [45]. Human fH bound well to all strains that expressed NspA (Figure 9A), but only weakly to strains that expressed fHbp but lacked NspA, which is consistent with the fH binding data present in Figure 6B. Very weak binding of chimpanzee fH to strains expressing NspA was also noted (Figure 9A). None of the strains tested bound rhesus fH when incubated with heat-inactivated rhesus sera (Figure 9A). The fHbp− NspA− strain showed barely detectable binding to human fH, and as expected, did not bind fH from the other primate species tested (Figure 9A).

### NspA expression enhances serum resistance and inhibits C3 deposition

**Figure 10:**
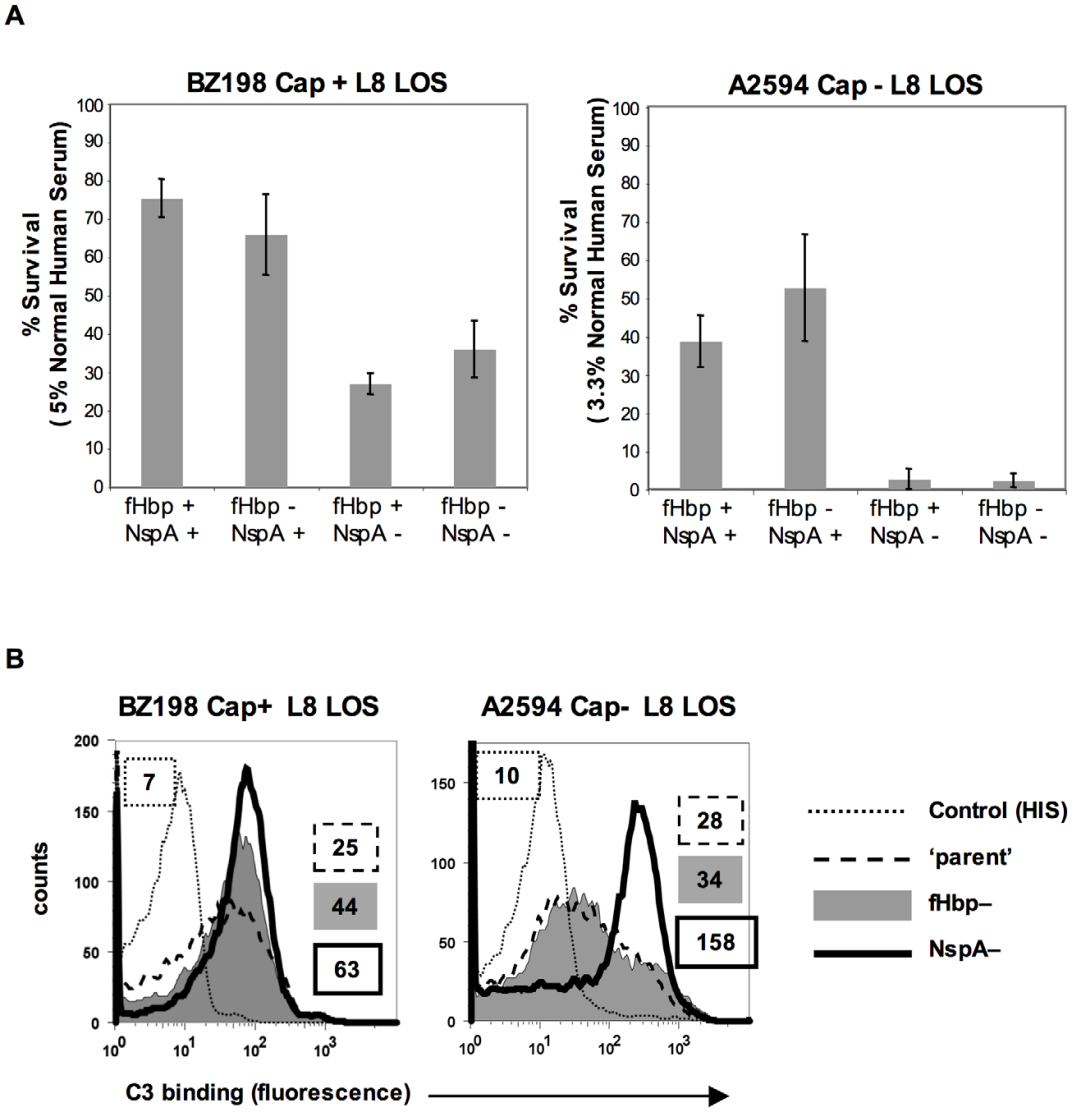
NspA expression enhances resistance of meningococci to complement-dependent killing and limits C3 deposition on bacteria. **A**. Strains BZ198 Cap+/L8 LOS and A2594 Cap−/L8 LOS and their isogenic mutant derivatives that lacked fHbp, NspA, or both fHbp and NspA were tested for their ability to resist killing by normal human serum in a serum bactericidal assay. The y-axis represents percent survival. Error bars indicate standard deviation calculated from 3 independent experiments. In all cases the decreased survival observed in strains that lack NspA was statistically significant (P<0.02 by a t-test) compared to the parent strain expressing NspA and fHbp **B**. C3 deposition on strains BZ198 Cap+/L8 LOS and A2594 Cap−/L8 LOS and their isogenic mutants that lacked either fHbp or NspA expression. The BZ198 Cap+ mutants were incubated with 40% NHS while the A2594 Cap− mutants were incubated with 20% NHS. C3 deposition on bacteria was detected by flow cytometry. Axes are as described for Figure 1A. Data with the double fHbp−/NspA− mutant was similar to the NspA− mutant and has been omitted for ease of visualization.

fH functions to down-regulate the alternative pathway of complement and bacteria that bind to fH would be expected to be more resistant to the bactericidal action of serum than those that do not bind to fH. To determine the relative roles of fHbp and NspA in serum resistance we examined strains BZ198 Cap+ and A2594 Cap− each expressing L8 LOS and their isogenic mutants that lacked either fHbp or NspA or both for their ability to resist killing by normal human serum. The concentration of serum used was determined based on the survival of each parent strain in serum (Supplementary Figure S5). Loss of NspA expression in both instances resulted in greater sensitivity to complement-dependent killing (Figure 10A). It is noteworthy that in these high NspA-expressing strains, deleting fHbp did not negatively impact survival. Deleting *fHbp* from the high fHbp-expressing strain H44/76, which expresses low levels of NspA, results in decreased serum resistance [13], [42].

fH limits C3 deposition by virtue of its ability to act as a cofactor in the factor I-mediated cleavage of C3b [15] and irreversibly dissociate alternative pathway C3 convertases (decay-accelerating activity) [16], [17]. As expected, mutant strains that lacked NspA bound more C3 than their NspA-sufficient ‘parent’ strains. The median fluorescence of C3 binding was ∼5-fold more with A2594 Cap−/L8 LOS/NspA− and ∼2.5-fold more with BZ198 Cap+/L8 LOS compared to their respective isogenic parent strains (Figure 10B). fH binding (Figure 5B) mirrored survival of bacteria in serum (Figure 10A) confirming that complement regulation by NspA occurred at the level of C3 deposition. Similarly, C3 deposition on BZ198 Cap+/LNT sia+/NspA− was ∼1.5-fold higher than on BZ198 Cap+/LNT sia+ (data not shown).

### Complementation of fHbp− NspA− double mutants with NspA restores binding of fH and serum resistance to meningococci

**Figure 11:**
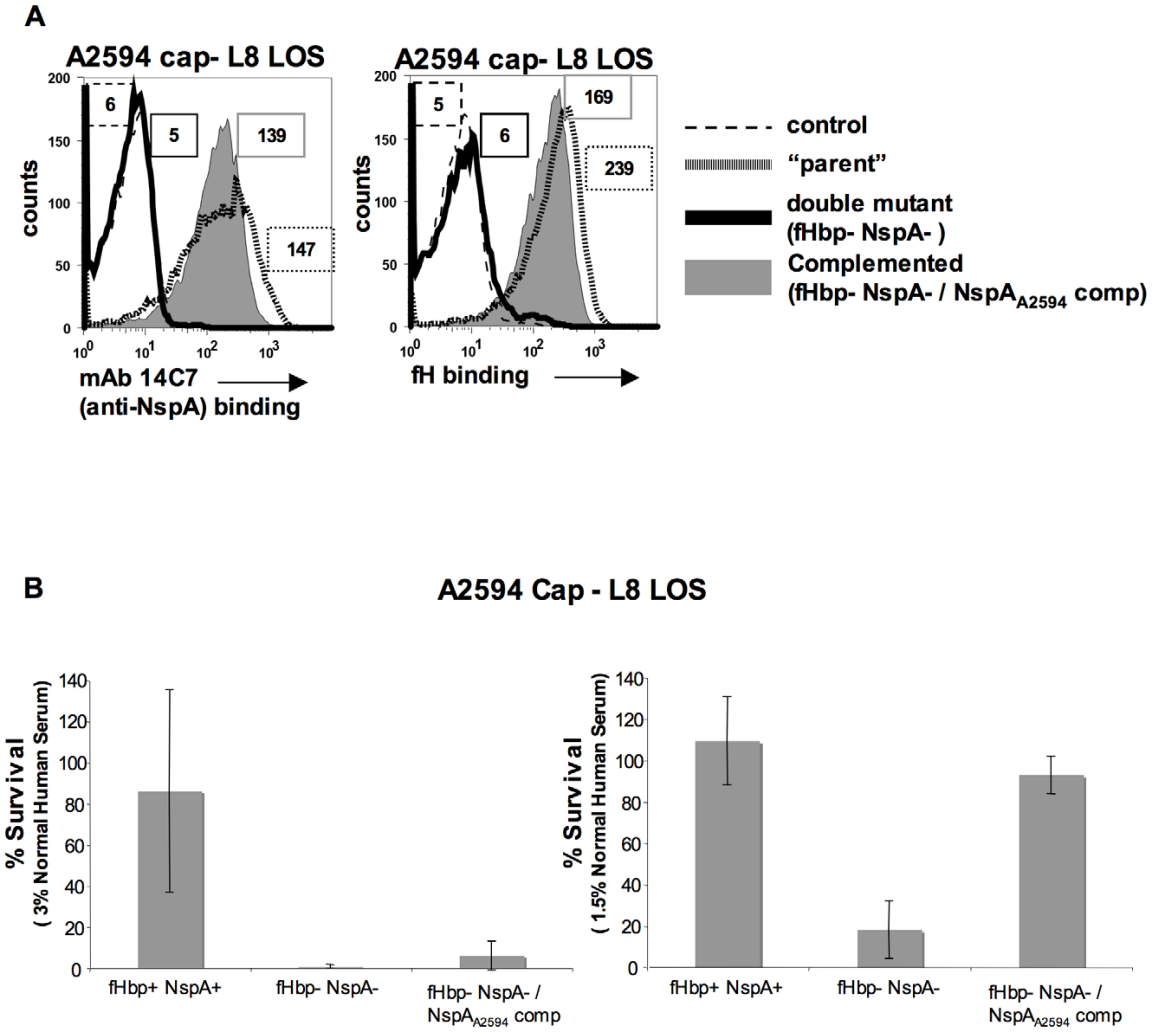
Complementation of fHbp NspA double mutants with NspA restores fH binding and serum resistance. **A**. Meningococcal strain A2594 Cap−/L8 LOS (“parent”), its *fHbp nspA* double mutant (fHbp− NspA−) and its *fHbp nspA* double mutant complemented with NspA (fHbp− NspA−/NspA_A2594_ comp) were examined for their ability to bind to MAb 14C7 (anti-NspA) and to fH (20 µg/ml) by flow cytometry. The boxed numbers accompanying each histogram represents the median fluorescence of 14C7 or fH binding to the entire bacterial population. Controls (shown by the broken lines) represent fluorescence where either mAb 14C7 or fH or was omitted from the reaction mixture. Axes are as described for Figure 1A. **B**. Strain A2594 Cap−/L8 LOS (fHbp+ NspA+), its *fHbp nspA* double mutant (fHbp− NspA−) and its *fHbp nspA* double mutant complemented with NspA (fHbp− NspA−/NspA_A2594_ comp) were tested for their ability to resist killing by NHS at concentrations of 3% (left graph) or 1.5% (right graph) in a serum bactericidal assay. The y-axis represents percent survival. Error bars indicate standard deviation calculated from 3 independent experiments. With 1.5% NHS the decreased survival observed in strains that lack NspA was statistically significant (P<0.02 by a t-test) compared to the parent strain expressing both NspA and fHbp.

Meningococcal strain A2594 Cap− L8 LOS lacking both fHbp and NspA was complemented, *in trans*, with NspA_A2594_ to verify that the loss in fH binding and concomitant decrease in serum resistance were not due to secondary changes. As expected, complementation with NspA_A2594_ resulted in expression of NspA as judged by both western blot (data not shown) and flow cytometry (Figure 11A). Restoration of NspA expression also restored the ability of fHbp− NspA− double mutants to bind fH (Figure 11A). The ability of the complemented strains to resist killing by NHS was assessed in a serum bactericidal assay. As shown above (Figure 10A), A2594 Cap− L8 LOS lacking both fHbp and NspA was more sensitive to serum killing than the parent strain expressing both of these proteins. Complementation with NspA, alone, restored serum resistance to the mutant strain lacking both fH ligands, albeit to levels less than that of the parent strain (Figure 11B). All three strains were completely (100%) killed in 6.6% NHS (data not shown). Overall, these data indicate that the lack of fH binding and decreased serum resistance observed in strains lacking NspA is because of lack of NspA expression and not the result of secondary changes in these isogenic strains.

## Discussion

Several pathogens, including bacteria, fungi, parasites and viruses bind to fH, which inhibits complement activation on their surface (reviewed in [49], [50]). This work has characterized NspA as a ligand for human fH and has shown that NspA plays a role in conferring serum resistance to meningococci even in the absence of expression of the previously characterized fH-binding meningococcal molecule, fHbp. NspA interacts with fH SCRs 6 and/or 7 and like fHbp, preferentially binds to human fH.

It is interesting that all naturally occurring meningococcal strains reported thus far express both fHbp [12], [51] and NspA [52], suggesting an important role for these proteins in meningococcal pathogenesis. Prior to this study the function of NspA had not been defined. The factors that influence fH binding to NspA on intact bacteria have been characterized in this study, which provides insights into the pathophysiological conditions or niches where NspA-mediated fH binding may assume an important role. Meningococcal strains that are isolated from the nasopharynx are often unencapsulated and/or express L8 LOS [53]; high binding of fH to NspA under these conditions could point to a key role for NspA in survival of meningococci during nasopharyngeal colonization (a prerequisite of invasive disease) and in survival of carrier strains. The positive effects of NspA on bacterial survival are also seen in encapsulated strains that are high NspA expressers such as BZ198 when they express L8 LOS (Figure 10A). Meningococcal isolates often express more than one LOS species because many of the genes involved in LOS biosynthesis, including *lgtA*, are phase variable [28]. Thus, for example, a strain could express a combination of LNT and L8 LOS species [54], [55]. It is not clear how LOS sialylation, which represents elongation beyond the LNT structure, enhances fH binding to NspA. One possibility is that LOS and NspA lie in close proximity and expression of the unsialylated LNT hinders fH from binding to NspA; sialylation may alter the conformation of LOS thereby better exposing the fH binding region of NspA. Another possibility is that LOS sialic acid itself may act as part of the docking site for fH. Nevertheless, LOS sialylation is not essential for fH binding to NspA on intact organisms. Sialylation of *N. gonorrhoeae* LNT LOS also enhances fH binding, but the interaction in that instance requires the concomitant presence of the gonococcal PorB molecule [56]. LPS glycan extensions can negatively impact binding of complement inhibitors to gram-negative bacteria. As an example, expression of O-antigenic repeats on the LPS of *Y. enterocolitica* can block binding of fH to the Ail protein [27]. Neisseriae lack O-antigenic repeats, yet subtle changes in the core LOS structure can have profound impacts on the binding of complement inhibitors and serum resistance. The presence of the proximal Glc off HepI appears to be necessary for optimal C4b-binding protein (C4BP) binding to porin (Por) B.1B (Por1B)-expressing gonococci [29].

NspA forms an eight-stranded anti-parallel β-barrel and has four putative surface exposed loops. A conformational epitope that includes NspA loop 3 appears to be important for binding of mAbs Me-7 [57] and 14C7 [40] both of which inhibit fH binding to NspA on meningococci. It is therefore possible that NspA loop 3 plays a role in the interaction with fH, although steric hindrance by the surface-bound mAb could account for the ability of the mAbs to block fH binding. It would be of interest to determine whether NspA plays a role in fH binding to gonococci because of the extensive (∼95%) sequence similarity between gonococcal and meningococcal NspA.

Sera from humans may contain naturally-occurring antibodies that are directed against LNT-expressing LOS and is bactericidal against group B meningococci [58]. Phase variation of *lgtA* that results in L8 LOS expression could subvert killing by these naturally occurring anti-LNT antibodies. However, truncation of the HepI chain of LOS could have a negative effect on serum resistance because of increased accessibility of the 3-phosphoethanolamine (PEA) residue on HepII to C4b [59]; C4b amide-linked to PEA can lead to downstream complement activation that may result in bacterial killing. By virtue of enhanced fH binding, high NspA expressers may be able to dampen excessive complement activation that is initiated by C4b when LOS is truncated.

It is noteworthy that loss of NspA (leaving fHbp intact) from a high NspA expressing strain such as A2594 (intermediate fHbp expression levels) resulted in increased C3 deposition on bacteria, while loss of fHbp (leaving NspA intact) in that strain did not enhance C3 deposition (Figure 10B). Strain BZ198 also expresses high levels of NspA and intermediate levels of fHbp; loss of NspA resulted in greater enhancement of C3 deposition relative to that seen when fHbp was deleted (Figure 10B). We have shown previously that loss of fHbp in high-fHbp expressing strains such as H44/76 (expresses low levels of NspA) also increases C3 deposition [13], [42]. The relative abilities of the two ligands to regulate C3 deposition on different strains may reflect heterogeneity in their expression levels. In addition, variables such as the amount of capsule expression and the diversity of HepI LOS extensions could affect the amount of fH binding to NspA and thereby its ability to regulate C3 deposition. The relative roles of fHbp and NspA in regulating complement activation in the context of expression of the different capsular groups and varying LOS structures is a complex subject that merits further study. However, it is evident from the current study and from previous work [13], [42], [60], [61] that both molecules contribute to the ability of meningococci to resist killing by normal human serum.

fHbp has shown considerable promise as a vaccine candidate [20]. A vaccine that has fHbp as a component could lead to selection of meningococcal strains that either do not express, or express very low amounts of fHbp. Under such circumstances, high NspA expressers may have a survival advantage. Our data suggest that including NspA as part of a vaccine strategy that targets fH-binding proteins on *N. meningitidis* could, in theory, overcome this potential obstacle. Indeed, NspA has been intensively investigated as a vaccine candidate against group B meningococci [62], [63]. Both mAbs against NspA (including Me-7 and 14C7) and polyclonal Abs against NspA (raised by immunization of mice with meningococcal outer membrane vesicles that contained native NspA) were bactericidal and protected against experimental murine infection [52], [64]. Although recombinant NspA expressed in *E. coli* and purified from inclusion bodies elicited a good antibody response in humans, these antibodies were not bactericidal [62]. Recombinant NspA does not have the same conformation as NspA present in the meningococcal outer membrane [40], [64], suggesting that protective antibodies may be directed against conformational epitopes. Another intriguing possibility, in light of our observations that binding of fH to NspA is restricted to humans, is that human fH may bind to NspA in the vaccine formulation, which could have attenuated the antibody response to surface-exposed (and fH binding) NspA epitopes that otherwise would have elicited a more productive bactericidal antibody response; this possibility has been raised previously with regard to the use of fH binding proteins as vaccines [14], [65].

In summary, we have identified an important complement-evasion function for NspA, an antigen that has been studied for its potential as a group B meningococcal vaccine candidate. In addition to the implications for fHbp-based vaccines that are currently being developed, these findings set the stage for further studies to characterize NspA-fH interactions that could boost efforts to develop better meningococcal vaccines.

## Materials and Methods

### Ethics statement

This study was approved by the Committee for the Protection of Human Subjects in Research at the University of Massachusetts Medical School. All subjects who donated blood for this study provided written informed consent.

### Bacterial strains, mutagenesis and bacterial growth conditions

The relevant phenotypes of the mutants created in *N. meningitidis* are listed in Table 1. The characteristics meningococcal strains used in this study are listed in Supplementary Table S2. Bacteria were routinely grown on chocolate agar plates supplemented with IsoVitaleX equivalent at 37°C in an atmosphere enriched with 5% CO_2_. GC plates supplemented with IsoVitaleX equivalent were used for antibiotic selection. Antibiotics where used at the following concentrations when indicated; 100 µg/ml kanamycin, 7 µg/ml chloramphenicol, 5 µg/ml erythromycin, 50 µg/ml spectinomycin and 5 µg/ml tetracycline. *Escherichia coli* strains (Invitrogen, Carlsbad, CA) were routinely cultured in Luria Bertani (LB) broth or on LB agar. Antibiotic were used as needed at the following concentrations: 50 µg/ml kanamycin, 150 µg/ml ampicillin, 50 µg/ml chloramphenicol, 400 µg/ml erythromycin, 100 µg/ml spectinomycin and 12.5 µg/ml tetracycline.

**Table 1:** Description of genotype and phenotype of mutations used in this study.

Mutation	Genotype	Phenotype
mynB	*mynB::Cm*	results in unencapsulated derivative of group A meningococci
fHbp	*fHbp::Erm*	fHbp not expressed
lgtA	*lgtA::Kan*	results in LOS with lactose substitution on HepI (Gal→Glc→HepI)
lgtF	*lgtF::Spc*	results in LOS without glycan extensions off HepI (HepI unsubstituted)
siaD	*siaD::Cm*;	interruption of the polysialyltransferase (*siaD*) in groups B, C, W-135 or Y results in unencapsulated mutants
siaA	*siaA::Cm*;	blocks first enzyme in sia operon (GlcNAc-6-P epimerase); results in unencapsulated strains that cannot synthesize CMP-NANA and therefore cannot endogenously sialylate its LNT LOS
lst	*lst::Kan*	interruption of the LOS sialyltransferase *(lst)* prevents LOS sialylation
nspA	*nspA::Spc*	No NspA expression
porB3	*porB3::Erm*	No PorB3 expression
porA	*porA::Kan*	No PorA expression
nspA++		*nspA* under control of *porA* promoter; high-level NspA expression
nspA_A2594_comp	*pFP12 NspA A2594 (Tet^R^)*	*nspA under the control of it's native promoter expressed from the low copy plasmid pFP12Tet*

Cm, chloramphenicol; Erm, erythromycin; Kan, kanamycin; Spc, specctinomycin; Tet, tetracycline.

Construction of *lgtA* [59], *lgtF* [59], *mynB* [56], *siaD* [59], *lst* [66] and *fHbp* [13] deletion mutants have all been described previously. Loss of capsule expression was verified by either colony hybridization or flow cytometry with the appropriate serogroup specific anti-capsule Ab. Anti-group A mAb JW-A-1 (IgG2a), anti-group C mAb KS-C-1 (IgG3), anti-group W-135 mAb JW-W1b (IgG2b) and anti-group Y mAb JW-Y2a (IgM) were provided by Dr. Dan M. Granoff (Childrens Hospital Oakland research Institute, Oakland, CA), while anti-group B mAb 2-2-B (IgM) was obtained from the National Institute for Biological Standards and Control (Potters Bar, Hertfordshire, U.K). In addition, inactivation of *siaD* (*mynB* in the case of group A) was verified by PCR. For serogroups A, C, Y and W-135 the confirmatory PCR was the amplification of a fragment corresponding to the predicted size of *siaD* (or *mynB* for group A) plus the resistance marker (∼2.5kb for serogroup A using primers NT2 5′-ATGATGGTAATGGGAAAAGAGT-3′ and NT4 5′-ATACTTAATAACAGAAAATGGCG-3′; ∼2.9 kb for serogroup C using primers BF 5′-AGCGTCAACGAATATGAAACATTAT-3′ and CR 5′-CTGCTTAACTTTATTAAGGGCATTG-3′ and ∼2.8 kb from serogroups W-135 and Y strains using primers W1618 5′-ATTCCCCATGAACTACATCAGAATA-3′ and W2766 5′-TAATGCAAACTCAATTGCAAAACTA-3′) coupled with the absence of a wildtype gene. In serogroup B strains *siaD* is inactivated with the 8.9 kb Tn1725 and a lack of amplification of the wild type *siaD*, in the presence of a positive control reaction, was used to demonstrate the lack of the wild type *siaD*. All Cap− serogroup B strains were verified by lack of reactivity to the anti-group B capsule mAb 2-2-B. LOS structure was verified in all strains and mutants by silver staining of protease K digested bacterial lysates that had been separated on 12% Bis-Tris gels (Invitrogen, Carlsbad, CA) using MES buffer (Invitrogen, Carlsbad, CA) as described previously [67]. In addition insertions in *lgtA* or *lgtE* were verified by PCR.

Mutant derivatives of strain A2594 that lacked PorA or PorB3 expression were constructed using DNA extracted from PorA and PorB3 deletion mutants in strain H44/76 (*porA::kan* and *porB3::erm*, respectively) that were provided by Dr. Peter Van der Ley (Laboratory of Vaccine Research, Netherlands Vaccine Institute, Bilthoven, The Netherlands).

NspA deletion mutants were constructed as follows. A 1.3 kb fragment of DNA containing *nspA* was amplified from *N. meningitidis* strain A2594 using the primers nspA_F114 (5′-CTCTTTAGGTTCTGCCAAAGGCTTC-3′) and nspA_R1122 (5′-ATGTTGTGAAGTGGGAAAGTGTTGC-3′) and the amplicon was cloned into pCR2.1-TOPO (Invitrogen, Carlsbad, CA). The resulting plasmid was digested with *Hinc*II, deleting an internal 130 bp fragment of *nspA*, and ligated to a blunt spectinomycin resistance cassette containing *aadA*. Linearized plasmid DNA was used to transform *N. meningitidis* strains as previously described [68]. PCR was used to confirm the *nspA::spc* genotype and Western blot using anti-NspA mAb Me-7 were performed to demonstrated loss of NspA.

A derivative of the *E. coli*-Neisseria shuttle vector pFP12 was used to complement the *nspA::spc* mutations *in trans*. A 1,131 bp fragment, containing *nspA* with its native promoter and terminator was amplified from chromosomal DNA prepared from strain A2594 using the primers NspA-R1213 StuI 5′-GAC**AGGCCT**GTTTTGGACATTTCGGATTCCTC-3′ and NspA-F102 SphI 5′-GAC**GCATGC**CACTATATAAGCGCAAACAAATCG-3′. The amplified DNA was digested with *StuI* and *SphI* and cloned into identically digested pFP12-GNA1870 [69]. The resulting construct was then digested with *ScaI* to allow for the insertion of a blunt (*BsrBI*) TetM cassette. The resulting plasmid construct, pFP12 NspA_A2594_Tet, was confirmed by DNA sequencing and by Western blot analysis of *E. coli* cell lysates using ME-7. A2594 Cap− L8 (Cm^R^, Kan^R^) and its fHbp− (erm^R^) and NspA− (spc^R^) and fHbp− NspA− double mutants were transformed with pFP12 NspA_A2594_Tet as described above. Tetracycline resistant transformants were screened by PCR and Western blot with Me-7. In addition the DNA sequence of the complementing *nspA* was verified.

Expression of NspA was up-regulated in some strains that naturally express low levels of NspA by replacing the *nspA* promoter with the promoter of *porA*. In brief, an approximately 200 bp fragment of DNA containing the promoter region of *porA* was amplified by PCR from genomic DNA isolated from group B strain M986 using the following primers: 5′-CTCATCGATGGGCAAACACCCGATACG-3′ (introducing site *Cla*I) and 5′-CTCACGCGTGAGGTCTGCGCTTGAATTGTG-3′ (introducing site *Mlu*I). This fragment was ligated into a 700 bp region upstream of *nspA* amplified by PCR from Z1092 genomic DNA using primers 5′-CATAAGCTTCGTAGCGGTATCCGGCTGC-3′ and 5′-CGCTGCCGAAGATTTGCCGGCAAATCTTCGGCAGCG-3′. This, in turn, was ligated into the *EcoR*I and *Hind*III sites of the cloning vector pGEM3zf(-) (Promega Corporation, Madison, Wisconsin). An erythromycin resistance cassette, *ermB*, was inserted upstream of the *porA* promoter within the fragment upstream of *nspA*. *N. meningitidis* strain Z1092 was transformed by adding plasmid DNA in 10 mM MgCl_2_ to colonies of Z1092 and incubating at 37°C enriched with 5% CO_2_ for 5 hours prior to plating onto chocolate agar with 5 µg/ml or erythromycin. The level of NspA expression in erythromycin-resistant colonies was analyzed by SDS-PAGE and Western blotting using murine antisera raised against His-tagged-NspA. All analyzed transformants expressed approximately 5-times the level of NspA when compared with the wild-type strain (data not shown). DNA extracted from Z1092 overexpressing NspA was used to transform strain Y2220 and Y2220/L8 LOS; colonies resistant to erythromycin were screened for increased NspA production compared to the parent strain by Western blotting.

*E. coli* BL21 (DE3) pGMS 1.0 harboring a functional copy of *nspA* and the preparation of microvesicles that contain NspA have been described previously [40].

For simplicity, the capsule and LOS phenotype of each mutant has been designated as follows: encapsulated strains, Cap+; unencapsulated mutants, Cap−; sialylated lacto-N-neotetraose LOS, LNT sia+; unsialylated lacto-N-neotetraose LOS, LNT; LOS with lactose extension off HepI (*lgtA* mutants), L8 LOS and LOS with no HepI saccharide extensions (*lgtF* mutants), HepI unsubstituted.

### Sera

Serum collected from a healthy human volunteer without a history of meningococcal disease and who had not received any meningococcal vaccines (normal human serum; NHS) was aliquoted and stored at −80°C till used. Hemolytic activity of the serum was confirmed using the Total Haemolytic Complement assay (Binding Site, Birmingham, U.K). Chimpanzee, baboon and rhesus sera were purchased from Bioreclamation (Bioreclamation, Hicksville, NY). Complement activity in the sera was destroyed by heating at 56°C for 30 minutes. The serum used did not contain any fHbp- or NspA-specific antibodies as revealed by western blots of whole bacterial lysates that were probed with serum.

### Flow cytometry

Flow cytometry to detect bound fH was performed as described previously [44]. Briefly, bacteria grown overnight on chocolate agar plates were washed with Hanks Balanced Salt Solution (HBSS) containing 1mM Ca^2+^ and 1 mM Mg^2+^ (HBSS^++^) and suspended to a final concentration of 3×10^8^ cells/ml; 10^8^ organisms were centrifuged and incubated with fH purified from human plasma (Complement Technology, Inc.; concentration specified for each experiment). Bound fH was detected using either affinity-isolated sheep anti-human fH (Lifespan Biosciences) or an anti-fH mAb (Quidel, catalog no. A254 (mAb 90×)), as available. While the polyclonal antibody provided higher sensitivity, relative differences in fH binding among strains using the two reagents were similar. FITC conjugated anti-sheep IgG or anti-mouse IgG (Sigma) were used as secondary antibodies. All reaction mixtures were carried out in HBSS^++^/1% BSA in a final volume of 50 µl. Flow cytometry was performed using a FACSCalibur instrument (Becton Dickinson) and data analysis was performed using the FlowJo data analysis software package (www.TreeStar.com).

FH / murine Fc fusion constructs that contain contiguous fH SCR domains (SCRs 1–5, 1–7, 6–10, 11–15, or 16–20) fused to the N-terminus of the Fc fragment of murine IgG2a (fH/Fc fusion proteins) have been described in detail previously [45]. To detect binding of recombinant fH/Fc fusion proteins, bacteria were incubated with concentrated tissue culture supernatant containing 0.5 µg of recombinant fH/Fc protein (as determined by ELISA) in a final reaction volume of 100 µl for 30 min at 37°C. After washing, FITC-labeled goat anti-mouse IgG (Sigma-Aldrich) diluted 1∶100 in 1% BSA/HBSS++ was used to detect bacteria-bound fH/Fc fusion proteins.

Recombinant Factor H-like protein-1 (FHL-1) was generated as previously described [44]. Following incubation of bacteria with 0.5 µg purified FHL-1, bound FHL-1 was detected using monoclonal (mAb) 90× (specific for SCR1; detects both full-length fH and FHL-1) and FITC-conjugated anti-mouse IgG (Sigma) as previously described [44].

In some experiments, mAbs against NspA (mAb Me-7; IgG2a [57] and 14C7 [40]) were used to block fH binding to intact bacteria; mAb P1.9 against outer membrane protein PorA of strain A2594 (National Institute of Biological Standards and Control) was used as a control. Bacteria were incubated with tissue culture supernatants containing mAbs Me-7 or P1.9 (the concentration of mAb in supernatants was estimated by western blotting where serial dilutions of the supernatants were compared against purified mouse IgG standards of the same subclass) or purified mAb 14C7 for 15 min at 37°C followed by addition of purified fH. The reaction mixture was incubated for an additional 15 min and bound fH was detected using sheep anti-human fH as described above.

C3 deposition on bacteria that were incubated with normal human serum (concentration specified for each experiment) was measured using FITC-conjugated sheep anti-human C3 (Biodesign/Meridian Life Science, Inc.) as described previously [13].

### Bacterial membrane preparations

Membranes were prepared from *N. meningitidis* strains A2594 and H44/76 and their fHbp deletion mutants as previously described [13], [70]. Briefly, bacteria harvested from five plates after overnight culture on chocolate agar were suspended in normal saline. Bacteria were washed, suspended in 5 ml of PBS containing 10 mM EDTA, and incubated at 60°C for 30 min. The bacterial suspensions were sheared by sequential passage through progressively smaller-gauge needles (18- through 25-gauge). The resultant suspension was centrifuged at 5000×*g* for 10 min at 4°C to separate any intact cells and debris. The supernatant was collected and ultracentrifuged at 80,000×*g* for 90 min at 4°C to yield a pellet that was enriched in outer membranes.

### Western blotting

Far Western blotting was used to assess fH binding to membrane preparations as described previously [13]. Membrane proteins were separated on a 4–12% Bis-Tris gel (Invitrogen Life Technologies) using MOPS running buffer. Proteins were transferred to polyvinylidene difluoride membranes (Millipore) and blocked with PBS-1% dry milk for 30 min at room temperature. Blocked membranes were incubated overnight at 4°C with fH (1 µg/ml in PBS-0.05% Tween 20). fH-binding proteins were detected using affinity-isolated sheep anti-human fH (1 µg/ml in PBS-0.05% Tween 20) and disclosed using anti-sheep IgG-alkaline phosphatase.

Western blotting was also used to assess NspA and fHbp expression. To detect NspA, membranes were probed with tissue culture supernatants that contained anti-NspA mAb Me-7 followed by anti-mouse IgG alkaline phosphatase. To detect fHbp (variant 1, 2 and 3), membranes were probed with rabbit polyclonal anti-serum that recognized variants 1, 2 and 3 fHbp diluted 1∶1000 in TBS 0.02% Tween 20 followed by anti-rabbit IgG alkaline phosphatase. To ensure equal loading across lanes, membranes were incised horizontally at the level of the ∼40–50 kD marker prior to the blocking step and stained with Coomassie blue (Imperial Protein Stain kit, Pierce).

Binding of human, chimpanzee and rhesus macaque fH to neisserial strains was measured by Western blotting as described previously [45]. Briefly, 10^8^ bacteria were suspended in HBSS^2+^ and incubated for 30 min at 37°C with 10% (v/v) heat-inactivated NHS or heat-inactivated chimpanzee or rhesus serum in a final reaction volume of 100 µl. Bacteria were washed three times in HBSS^2+^, the bacterial pellets were lysed with lithium dodecyl sulfate sample buffer (Invitrogen, Carlsbad CA) and liberated fH was detected after electrophoresis and transfer to PVDF using goat polyclonal anti-human fH (Bethyl Laboratories, Montgomery, TX) that also recognizes chimpanzee and rhesus fH [45], followed by alkaline phosphatase-conjugated anti-goat IgG (Sigma). Human and the non-human primate sera alone served as positive controls.

### MALDI-TOF analysis

Outer membrane proteins were separated by electrophoresis as described above and stained with colloidal Coomassie brilliant blue (Sigma-Aldrich). The band corresponding to the ∼17 kDa band that bound fH was excised, washed extensively and then digested “in gel” with trypsin as described elsewhere [71]. Digested peptides were further purified via micro Zip Tipping. Briefly, samples dried down to a 10 µl volume were acidified with 1–2 µl of 1% TFA and then loaded on a Zip Tipμ-C18 (Millipore, Corp) that had been pre-equilibrated with 0.1% TFA. After washing with twice with 10 µl aliquots of 0.1%, TFA samples were deposited directly onto the MALDI sample target using 1 µl of Matrix solution (15 mg/ml of 2,5-dihydroxybenzoic Acid (MassPrep DHB, Waters Corp.) in 50∶50 acetonitrile: 0.1% TFA). Samples were allowed to air dry prior to insertion into the mass spectrometer. Analysis was performed on a Kratos Axima QIT (Shimadzu Instruments) matrix-assisted-laser desorption/ionization (MALDI) mass spectrometer. Peptides were analyzed in positive ion mode in mid-mass range (700–3000 Da). The instrument was externally calibrated with Angiotensin II (1046.54 Da), P14R (1533.86 Da) and ACTH (18–39) (2465.20 Da). Precursors were selected based on signal intensity at a mass resolution width of 250 for CID fragmentation using Argon as the collision gas. Database searches were performed in house with Mascot (Matrix Sciences, Ltd.) using the Peptide Mass Fingerprint program for MS data and the MS/MS Ion Search program for CID data. All identifications were confirmed or established with CID (MS/MS) data.

### Serum bactericidal assays

Susceptibility of meningococci to complement mediated killing was determined using a serum bactericidal assay as described previously [44], [72]. The optimal concentration of serum was determined empirically for each strain (Supplementary Figure S5). Bacteria from an overnight culture on chocolate agar plates were inoculated onto fresh chocolate agar and allowed to grow for ∼6 h at 37°C in 5% CO_2_. Normal human serum was obtained from a healthy human volunteer and stored at −70°C till used in bactericidal assays. Briefly, 2000 CFUs of meningococci were incubated with serum (concentrations specified for each experiment) in a final reaction volume of 150 µl. Aliquots of 25 µl were plated in duplicate at the start of the assay (*t*_0_) and after incubating the reaction mixture at 37°C for 30 min (*t*_30_). Survival was calculated as the number of viable colonies at *t*_30_ relative to baseline colony counts at *t*_0_. Each experiment was repeated at least three times.

### ELISA to detect binding of fH to microvesicles containing NspA

*E. coli* BL21(DE3) (Invitrogen, Carlsbad, CA) harboring recombinant NspA on plasmid pGMS 1.0 and *E. coli* BL21(DE3) transformed with pBluescript II SK+ (Stratagene, La Jolla, CA) were used to prepare microvesicles as previously described [40]. An ELISA was used to detect fH binding to NspA containing vesicles. Microtiter wells were coated with either NspA-producing vesicles or with control vesicles each at a concentration of 10 µg/ml in PBS overnight at 22°C. Nonspecific biding sites were blocked with PBS/2.5% BSA for 2 h at 37°C. To demonstrate the ability of anti-NspA mAb 14C7 to block fH binding to NspA-containing vesicles, select wells were incubated with mAb 14C7 (10 µg/ml) in PBS/0.05% Tween 20 for 1 h at 37°C; the remaining wells were incubated with PBS/Tween alone. fH (concentrations ranging from 0 to 10 µg/ml) was then added to wells for 1 h at 37°C, and bound fH was detected using polyclonal sheep anti-human fH followed by anti-sheep IgG conjugated with alkaline phosphatase, each for 1 h at 37°C.

### Statistical methods

Cuzick's nonparametric test for trend across ordered groups [73] was used to determine if there was a trend between the binding of fH and the length of glycan extensions from the HepI chain of LOS. Median fluorescence values from three independent experiments were used in the analysis. Strains expressing LNT LOS, L8 LOS and unsubstituted LOS were ordered decreasingly by the length of the HepI glycans extensions and scored as 5, 3 and 1, respectively. The measurement of binding was divided by the value of the control for normalizing. The analysis was done separately for strains A2594 Cap+ and A2594 Cap−. The results showed a statistically significant trend between fH binding and decreasing length of HepI glycan extensions for both Cap+ and Cap− strains (Table S2; results are the same for A2594 Cap+ and A2594 Cap−, p = 0.007).

For bactericidal assays the average survival was calculated from at least three independent experiments and error bars represent the standard deviation. A t-test was used to determine significance.

## Supporting Information

Figure S1 Neisserial Opa proteins are not involved in binding of fH to *N. meningitidis*. fH binding to Opa+ unencapsulated meningococcal strain Z2087 (solid black line) and its unencapsulated isogenic Opa negative mutant (shaded grey) was examined by flow cytometry. Bacteria were incubated with purified human fH at a concentration of 20 µg/ml and bound fH was detected with polyclonal sheep anti-human fH. Representative controls with the parent strain where fH was omitted from the reaction mixture is shown by the broken line. The x-axis represents fluorescence on a log_10_ scale and the y axis is the number of events.
(0.13 MB TIF)

Figure S2 Expression of fHbp and NspA in BZ198, A2594 and Z2087 derivatives as determined by Western blotting of whole cell lysates followed by detection with polyclonal anti-fHbp (variant 1,2 and 3) or anti-NspA mAb Me-7 as indicated. Strains with altered capsule (cap+ or cap−) and LOS structures were examines. The HepI of LOS was substituted with either lacto-N-neotetraose (LNT), lactose (L8) or was unsubstituted (U). Growth in the presence of CMP-NANA to sialylate LNT LOS is as indicated. After transfer, proteins migrating above ∼50 kD were stained with Coomassie blue and served as a loading control, proteins migrating between ∼20 kD and 40 kD were probed to detect fHbp and proteins migrating below 20 kD were probed to detect NspA. NspA migrates with an apparent molecular mass of approximately 15 kD when 4–12% Bis-Tris gels are used with MES running buffer. Of note, NspA is a heat-modifiable protein and the second larger anti-NspA-reactive band seen in some lanes is the result of incomplete heat denaturation.
(0.84 MB TIF)

Figure S3 Truncating the HepI chain of LOS in low and intermediate NspA expressing strains discloses fH binding. fH (10 µg/ml) binding to the Cap−/LNT sia− mutants of strains H44/76, C2120, W171 and Y2220 were compared to their isogenic mutants that lacked glycan extensions from HepI (HepI unsubstituted).
(0.28 MB TIF)

Figure S4 Binding of purified recombinant FHL-1 (7µg/ml) to encapsulated (gray shaded histogram) and unencapsulated (solid black line) *N. meningitidis* strain A2594 that expresses NspA but not fHbp. In all graphs, the x-axis represents fluorescence on a log_10_ scale and the y-axis the number of events. Numbers represent the median fluorescence of the corresponding histogram. Purified FHL-1 was omitted from control reaction mixtures (broken line).
(0.11 MB TIF)

Figure S5 Titration of serum concentrations to determine the level of serum resistance of BZ198 Cap+ L8 LOS and A2594 Cap− L8 LOS. Strains BZ198 Cap+ L8 LOS (left graph) and A2594 Cap− L8 LOS (right graph) were tested for their ability to resist killing by normal human serum in a serum bactericidal assay. The y-axis represents percent survival and the x-axis represents the percent serum used in the assay. Error bars indicate standard deviation calculated from 3 independent experiments.
(0.15 MB TIF)

Table S1 Estimated mean of normalized fH binding and p-values for trend test.
(0.05 MB DOC)

Table S2 Meningococcal strains used in this study and their relevant characteristics.
(0.15 MB DOC)

## References

[1] Goldschneider I, Gotschlich EC, Artenstein MS (1969). Human immunity to the meningococcus. I. The role of humoral antibodies.. J Exp Med.

[2] Figueroa J, Andreoni J, Densen P (1993). Complement deficiency states and meningococcal disease.. Immunol Res.

[3] Figueroa JE, Densen P (1991). Infectious diseases associated with complement deficiencies.. Clin Microbiol Rev.

[4] Fijen CA, Kuijper EJ, te Bulte MT, Daha MR, Dankert J (1999). Assessment of complement deficiency in patients with meningococcal disease in The Netherlands.. Clin Infect Dis.

[5] Ross SC, Densen P (1984). Complement deficiency states and infection: epidemiology, pathogenesis and consequences of neisserial and other infections in an immune deficiency.. Medicine (Baltimore).

[6] Jarvis GA, Vedros NA (1987). Sialic acid of group B *Neisseria meningitidis* regulates alternative complement pathway activation.. Infect Immun.

[7] Uria MJ, Zhang Q, Li Y, Chan A, Exley RM (2008). A generic mechanism in *Neisseria meningitidis* for enhanced resistance against bactericidal antibodies.. J Exp Med.

[8] Sa ECC, Griffiths NJ, Virji M (2010). Neisseria meningitidis Opc invasin binds to the sulphated tyrosines of activated vitronectin to attach to and invade human brain endothelial cells.. PLoS Pathog.

[9] Virji M, Griffiths NJ, van Alphen L, van der Ley P, van den Dobbelsteen G Binding of Opc to vitronectin contributes to increased serum resistance of *Neisseria meningitidis* isolates..

[10] Jarva H, Ram S, Vogel U, Blom AM, Meri S (2005). Binding of the complement inhibitor C4bp to serogroup B Neisseria meningitidis.. J Immunol.

[11] Fletcher LD, Bernfield L, Barniak V, Farley JE, Howell A (2004). Vaccine potential of the Neisseria meningitidis 2086 lipoprotein.. Infect Immun.

[12] Masignani V, Comanducci M, Giuliani MM, Bambini S, Adu-Bobie J (2003). Vaccination against Neisseria meningitidis using three variants of the lipoprotein GNA1870.. J Exp Med.

[13] Madico G, Welsch JA, Lewis LA, McNaughton A, Perlman DH (2006). The meningococcal vaccine candidate GNA1870 binds the complement regulatory protein factor H and enhances serum resistance.. J Immunol.

[14] Schneider MC, Prosser BE, Caesar JJ, Kugelberg E, Li S (2009). Neisseria meningitidis recruits factor H using protein mimicry of host carbohydrates.. Nature.

[15] Pangburn MK, Schreiber RD, Muller-Eberhard HJ (1977). Human complement C3b inactivator: isolation, characterization, and demonstration of an absolute requirement for the serum protein beta1H for cleavage of C3b and C4b in solution.. J Exp Med.

[16] Weiler JM, Daha MR, Austen KF, Fearon DT (1976). Control of the amplification convertase of complement by the plasma protein beta1H.. Proc Natl Acad Sci U S A.

[17] Whaley K, Ruddy S (1976). Modulation of the alternative complement pathways by beta 1 H globulin.. J Exp Med.

[18] Beernink PT, Granoff DM (2009). The modular architecture of meningococcal factor H-binding protein.. Microbiology.

[19] Pajon R, Beernink PT, Harrison LH, Granoff DM (2009). Frequency of factor H-binding protein modular groups and susceptibility to cross-reactive bactericidal activity in invasive meningococcal isolates.. Vaccine.

[20] Granoff DM (2010). Review of group B meningococcal vaccines.. Clin Infect Dis.

[21] Hammerschmidt S, Muller A, Sillmann H, Muhlenhoff M, Borrow R (1996). Capsule phase variation in Neisseria meningitidis serogroup B by slipped-strand mispairing in the polysialyltransferase gene (siaD): correlation with bacterial invasion and the outbreak of meningococcal disease.. Mol Microbiol.

[22] Deghmane AE, Giorgini D, Larribe M, Alonso JM, Taha MK (2002). Down-regulation of pili and capsule of Neisseria meningitidis upon contact with epithelial cells is mediated by CrgA regulatory protein.. Mol Microbiol.

[23] Claus H, Maiden MC, Maag R, Frosch M, Vogel U (2002). Many carried meningococci lack the genes required for capsule synthesis and transport.. Microbiology.

[24] Claus H, Maiden MC, Wilson DJ, McCarthy ND, Jolley KA (2005). Genetic analysis of meningococci carried by children and young adults.. J Infect Dis.

[25] Yazdankhah SP, Caugant DA (2004). Neisseria meningitidis: an overview of the carriage state.. J Med Microbiol.

[26] Schneider MC, Exley RM, Chan H, Feavers I, Kang YH (2006). Functional significance of factor H binding to Neisseria meningitidis.. J Immunol.

[27] Biedzka-Sarek M, Jarva H, Hyytiainen H, Meri S, Skurnik M (2008). Characterization of complement factor H binding to Yersinia enterocolitica serotype O:3.. Infect Immun.

[28] Berrington AW, Tan YC, Srikhanta Y, Kuipers B, van der Ley P (2002). Phase variation in meningococcal lipooligosaccharide biosynthesis genes.. FEMS Immunol Med Microbiol.

[29] Ram S, Ngampasutadol J, Cox AD, Blom AM, Lewis LA (2007). Heptose I glycan substitutions on Neisseria gonorrhoeae lipooligosaccharide influence C4b-binding protein binding and serum resistance.. Infect Immun.

[30] Ram S, Sharma AK, Simpson SD, Gulati S, McQuillen DP (1998). A novel sialic acid binding site on factor H mediates serum resistance of sialylated *Neisseria gonorrhoeae*.. J Exp Med.

[31] Madico G, Ngampasutadol J, Gulati S, Vogel U, Rice PA (2007). Factor H Binding and Function in Sialylated Pathogenic Neisseriae is Influenced by Gonococcal, but Not Meningococcal, Porin.. J Immunol.

[32] Mandrell RE, Kim JJ, John CM, Gibson BW, Sugai JV (1991). Endogenous sialylation of the lipooligosaccharides of *Neisseria meningitidis*.. J Bacteriol.

[33] Kogan G, Uhrin D, Brisson JR, Jennings HJ (1997). Structural basis of the Neisseria meningitidis immunotypes including the L4 and L7 immunotypes.. Carbohydr Res.

[34] Tsang RS, Law DK, Tsai C, Ng L (2001). Detection of the lst gene in different serogroups and LOS immunotypes of Neisseria meningitidis.. FEMS Microbiol Lett.

[35] Estabrook MM, Jack DL, Klein NJ, Jarvis GA (2004). Mannose-binding lectin binds to two major outer membrane proteins, opacity protein and porin, of Neisseria meningitidis.. J Immunol.

[36] Derrick JP, Urwin R, Suker J, Feavers IM, Maiden MC (1999). Structural and evolutionary inference from molecular variation in Neisseria porins.. Infect Immun.

[37] Feavers IM, Suker J, McKenna AJ, Heath AB, Maiden MC (1992). Molecular analysis of the serotyping antigens of Neisseria meningitidis.. Infect Immun.

[38] Suker J, Feavers IM, Achtman M, Morelli G, Wang JF (1994). The porA gene in serogroup A meningococci: evolutionary stability and mechanism of genetic variation.. Mol Microbiol.

[39] Urwin R, Fox AJ, Musilek M, Kriz P, Maiden MC (1998). Heterogeneity of the PorB protein in serotype 22 Neisseria meningitidis.. J Clin Microbiol.

[40] Hou VC, Moe GR, Raad Z, Wuorimaa T, Granoff DM (2003). Conformational epitopes recognized by protective anti-neisserial surface protein A antibodies.. Infect Immun.

[41] Moe GR, Tan S, Granoff DM (1999). Differences in surface expression of NspA among Neisseria meningitidis group B strains.. Infect Immun.

[42] Welsch JA, Ram S, Koeberling O, Granoff DM (2008). Complement-dependent synergistic bactericidal activity of antibodies against factor H-binding protein, a sparsely distributed meningococcal vaccine antigen.. J Infect Dis.

[43] Ripoche J, Day AJ, Harris TJ, Sim RB (1988). The complete amino acid sequence of human complement factor H.. Biochem J.

[44] Shaughnessy J, Lewis LA, Jarva H, Ram S (2009). Functional comparison of the binding of factor H short consensus repeat 6 (SCR 6) to factor H binding protein from Neisseria meningitidis and the binding of factor H SCR 18 to 20 to Neisseria gonorrhoeae porin.. Infect Immun.

[45] Ngampasutadol J, Ram S, Gulati S, Agarwal S, Li C (2008). Human Factor H Interacts Selectively with Neisseria gonorrhoeae and Results in Species-Specific Complement Evasion.. J Immunol.

[46] Friese MA, Hellwage J, Jokiranta TS, Meri S, Peter HH (1999). FHL-1/reconectin and factor H: two human complement regulators which are encoded by the same gene are differently expressed and regulated.. Mol Immunol.

[47] Granoff DM, Welsch JA, Ram S (2009). Binding of complement factor H (fH) to Neisseria meningitidis is specific for human fH and inhibits complement activation by rat and rabbit sera.. Infect Immun.

[48] Ngampasutadol J, Ram S, Blom AM, Jarva H, Jerse AE (2005). Human C4b-binding protein selectively interacts with Neisseria gonorrhoeae and results in species-specific infection.. Proc Natl Acad Sci U S A.

[49] Blom AM, Hallstrom T, Riesbeck K (2009). Complement evasion strategies of pathogens-acquisition of inhibitors and beyond.. Mol Immunol.

[50] Kraiczy P, Wurzner R (2006). Complement escape of human pathogenic bacteria by acquisition of complement regulators.. Mol Immunol.

[51] Murphy E, Andrew L, Lee KL, Dilts DA, Nunez L (2009). Sequence diversity of the factor H binding protein vaccine candidate in epidemiologically relevant strains of serogroup B Neisseria meningitidis.. J Infect Dis.

[52] Martin D, Cadieux N, Hamel J, Brodeur BR (1997). Highly conserved Neisseria meningitidis surface protein confers protection against experimental infection.. J Exp Med.

[53] Jones DM, Borrow R, Fox AJ, Gray S, Cartwright KA (1992). The lipooligosaccharide immunotype as a virulence determinant in Neisseria meningitidis.. Microb Pathog.

[54] Mackinnon FG, Borrow R, Gorringe AR, Fox AJ, Jones DM (1993). Demonstration of lipooligosaccharide immunotype and capsule as virulence factors for Neisseria meningitidis using an infant mouse intranasal infection model.. Microb Pathog.

[55] McLeod Griffiss J, Brandt BL, Saunders NB, Zollinger W (2000). Structural relationships and sialylation among meningococcal L1, L8, and L3,7 lipooligosaccharide serotypes.. J Biol Chem.

[56] Madico G, Ram S, Getzlaff S, Prasad A, Gulati S

[57] Cadieux N, Plante M, Rioux CR, Hamel J, Brodeur BR (1999). Bactericidal and cross-protective activities of a monoclonal antibody directed against Neisseria meningitidis NspA outer membrane protein.. Infect Immun.

[58] Estabrook MM, Jarvis GA, McLeod Griffiss J (2007). Affinity-purified human immunoglobulin G that binds a lacto-N-neotetraose-dependent lipooligosaccharide structure is bactericidal for serogroup B Neisseria meningitidis.. Infect Immun.

[59] Ram S, Cox AD, Wright JC, Vogel U, Getzlaff S (2003). Neisserial lipooligosaccharide is a target for complement component C4b: Inner core phosphoethanolamine residues define C4b linkage specificity.. J Biol Chem.

[60] Seib KL, Oriente F, Adu-Bobie J, Montanari P, Ferlicca F (2010). Influence of serogroup B meningococcal vaccine antigens on growth and survival of the meningococcus in vitro and in ex vivo and in vivo models of infection.. Vaccine.

[61] Seib KL, Serruto D, Oriente F, Delany I, Adu-Bobie J (2009). Factor H-binding protein is important for meningococcal survival in human whole blood and serum and in the presence of the antimicrobial peptide LL-37.. Infect Immun.

[62] Halperin SA, Langley JM, Smith B, Wunderli P, Kaufman L (2007). Phase 1 first-in-human studies of the reactogenicity and immunogenicity of a recombinant meningococcal NspA vaccine in healthy adults.. Vaccine.

[63] Martin D, Brodeur BR, Hamel J, Couture F, de Alwis U (2000). Candidate Neisseria meningitidis NspA vaccine.. J Biotechnol.

[64] Moe GR, Zuno-Mitchell P, Hammond SN, Granoff DM (2002). Sequential immunization with vesicles prepared from heterologous Neisseria meningitidis strains elicits broadly protective serum antibodies to group B strains.. Infect Immun.

[65] Meri S, Jordens M, Jarva H (2008). Microbial complement inhibitors as vaccines.. Vaccine.

[66] Vogel U, Claus H, Heinze G, Frosch M (1999). Role of lipopolysaccharide sialylation in serum resistance of serogroup B and C meningococcal disease isolates.. Infect Immun.

[67] Gulati S, Cox A, Lewis LA, Michael FS, Li J (2005). Enhanced factor H binding to sialylated Gonococci is restricted to the sialylated lacto-N-neotetraose lipooligosaccharide species: implications for serum resistance and evidence for a bifunctional lipooligosaccharide sialyltransferase in Gonococci.. Infect Immun.

[68] Lewis LA, Gipson M, Hartman K, Ownbey T, Vaughn J (1999). Phase variation of HpuAB and HmbR, two distinct haemoglobin receptors of Neisseria meningitidis DNM2.. Mol Microbiol.

[69] Hou VC, Koeberling O, Welsch JA, Granoff DM (2005). Protective antibody responses elicited by a meningococcal outer membrane vesicle vaccine with overexpressed genome-derived neisserial antigen 1870.. J Infect Dis.

[70] Gnehm HE, Pelton SI, Gulati S, Rice PA (1985). Characterization of antigens from nontypable *Haemophilus influenzae* recognized by human bactericidal antibodies. Role of Haemophilus outer membrane proteins.. J Clin Invest.

[71] Lahm HW, Langen H (2000). Mass spectrometry: a tool for the identification of proteins separated by gels.. Electrophoresis.

[72] McQuillen DP, Gulati S, Rice PA (1994). Complement-mediated bacterial killing assays.. Methods Enzymol.

[73] Cuzick J (1985). A Wilcoxon-type test for trend.. Stat Med.

