# Supplementary figures and images for: The Meningococcal Vaccine Candidate Neisserial Surface Protein A (NspA) Binds to Factor H and Enhances Meningococcal Resistance to Complement
Source: PLoS Pathog. 2010 Jul 29;6(7):e1001027. doi: 10.1371/journal.ppat.1001027 (PMC2912398; doi:10.1371/journal.ppat.1001027)

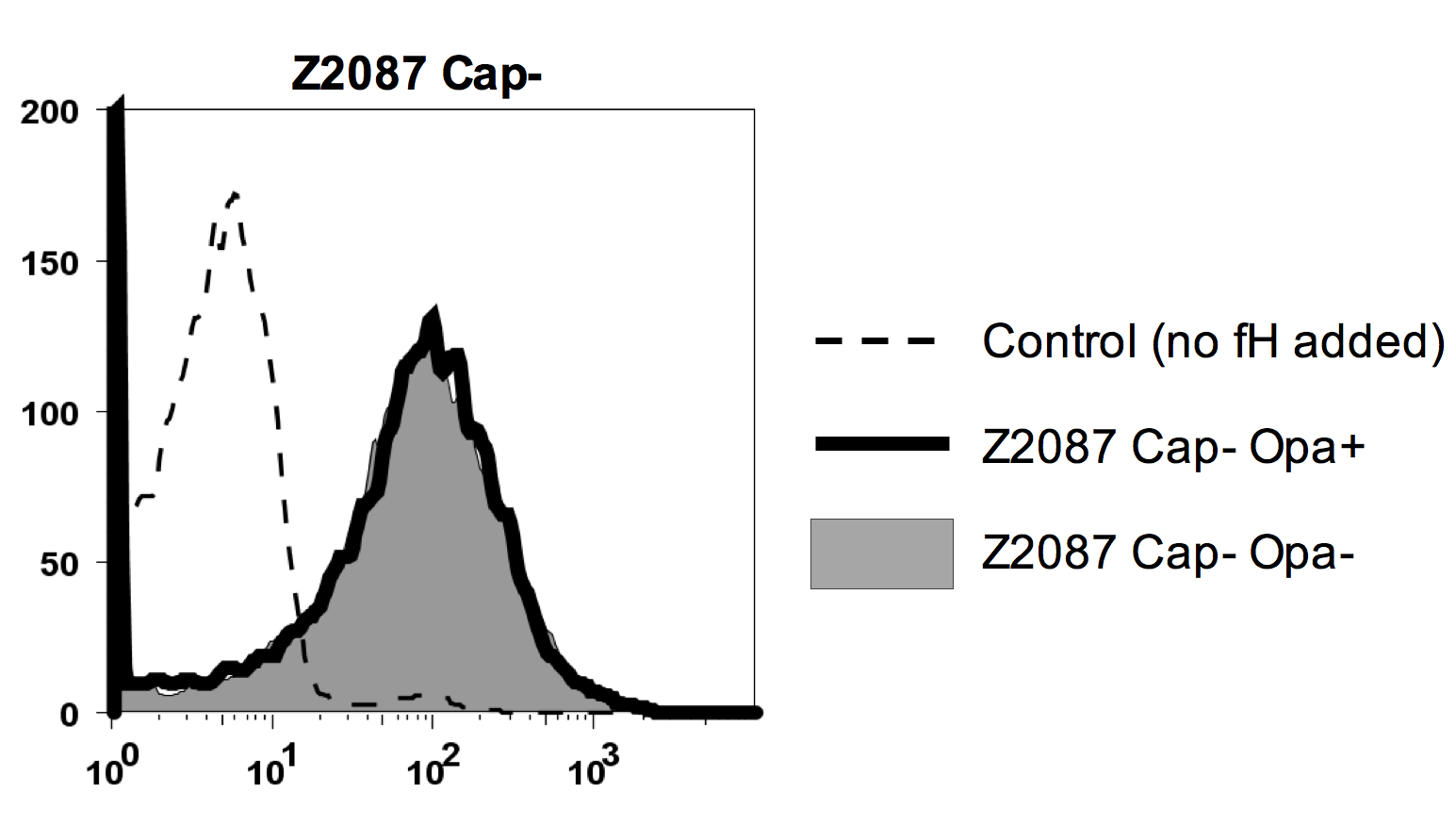

Supplement: Figure S1 — Neisserial Opa proteins are not involved in binding of fH to N. meningitidis. fH binding to Opa+ unencapsulated meningococcal strain Z2087 (solid black line) and its unencapsulated isogenic Opa negative mutant (shaded grey) was examined by flow cytometry. Bacteria were incubated with purified human fH at a concentration of 20 µg/ml and bound fH was detected with polyclonal sheep anti-human fH. Representative controls with the parent strain where fH was omitted from the reaction mixture is shown by the broken line. The x-axis represents fluorescence on a log10 scale and the y axis is the number of events. (0.13 MB TIF) [file ppat.1001027.s001.tif]

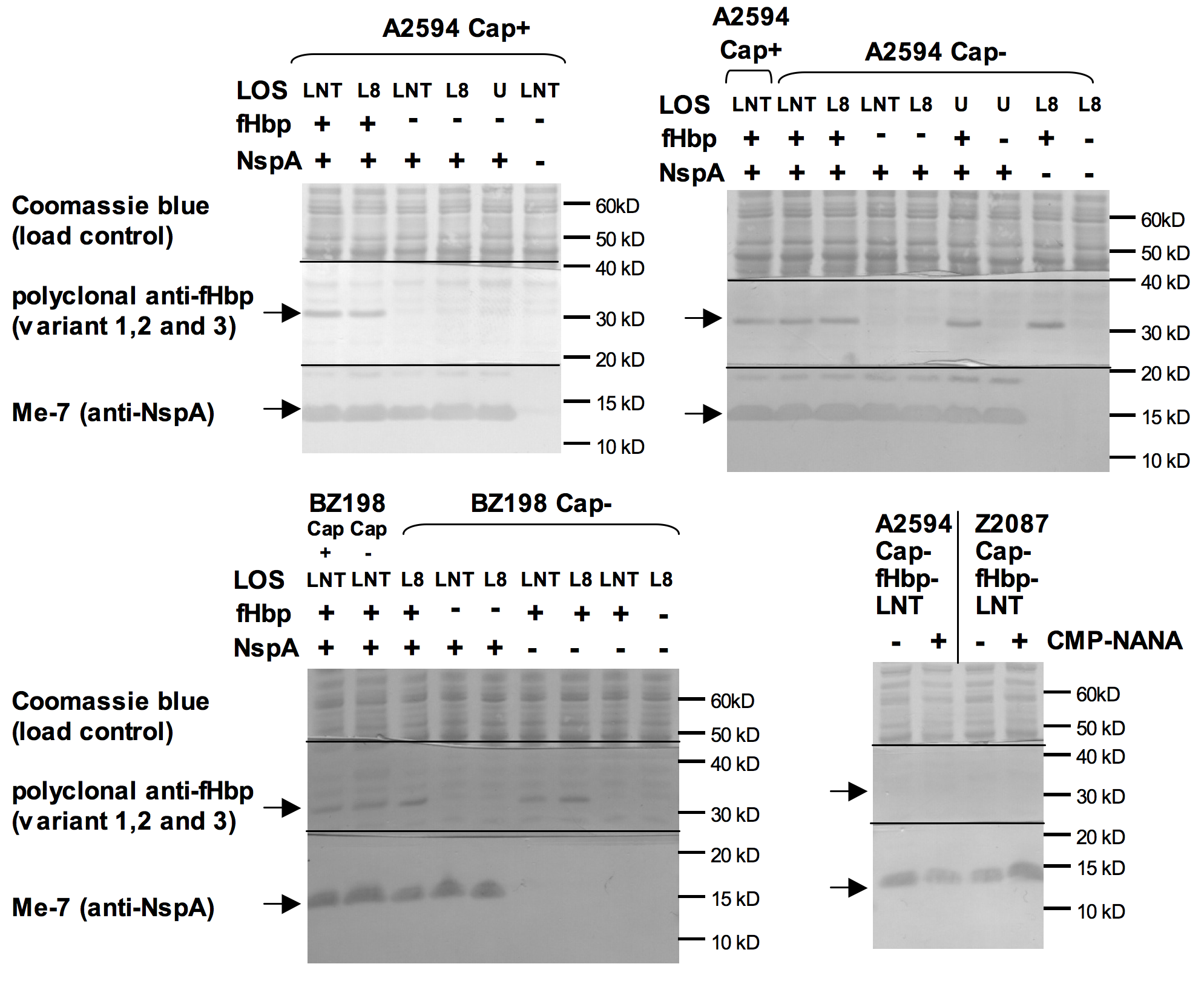

Supplement: Figure S2 — Expression of fHbp and NspA in BZ198, A2594 and Z2087 derivatives as determined by Western blotting of whole cell lysates followed by detection with polyclonal anti-fHbp (variant 1,2 and 3) or anti-NspA mAb Me-7 as indicated. Strains with altered capsule (cap+ or cap−) and LOS structures were examines. The HepI of LOS was substituted with either lacto-N-neotetraose (LNT), lactose (L8) or was unsubstituted (U). Growth in the presence of CMP-NANA to sialylate LNT LOS is as indicated. After transfer, proteins migrating above ∼50 kD were stained with Coomassie blue and served as a loading control, proteins migrating between ∼20 kD and 40 kD were probed to detect fHbp and proteins migrating below 20 kD were probed to detect NspA. NspA migrates with an apparent molecular mass of approximately 15 kD when 4–12% Bis-Tris gels are used with MES running buffer. Of note, NspA is a heat-modifiable protein and the second larger anti-NspA-reactive band seen in some lanes is the result of incomplete heat denaturation. (0.84 MB TIF) [file ppat.1001027.s002.tif]

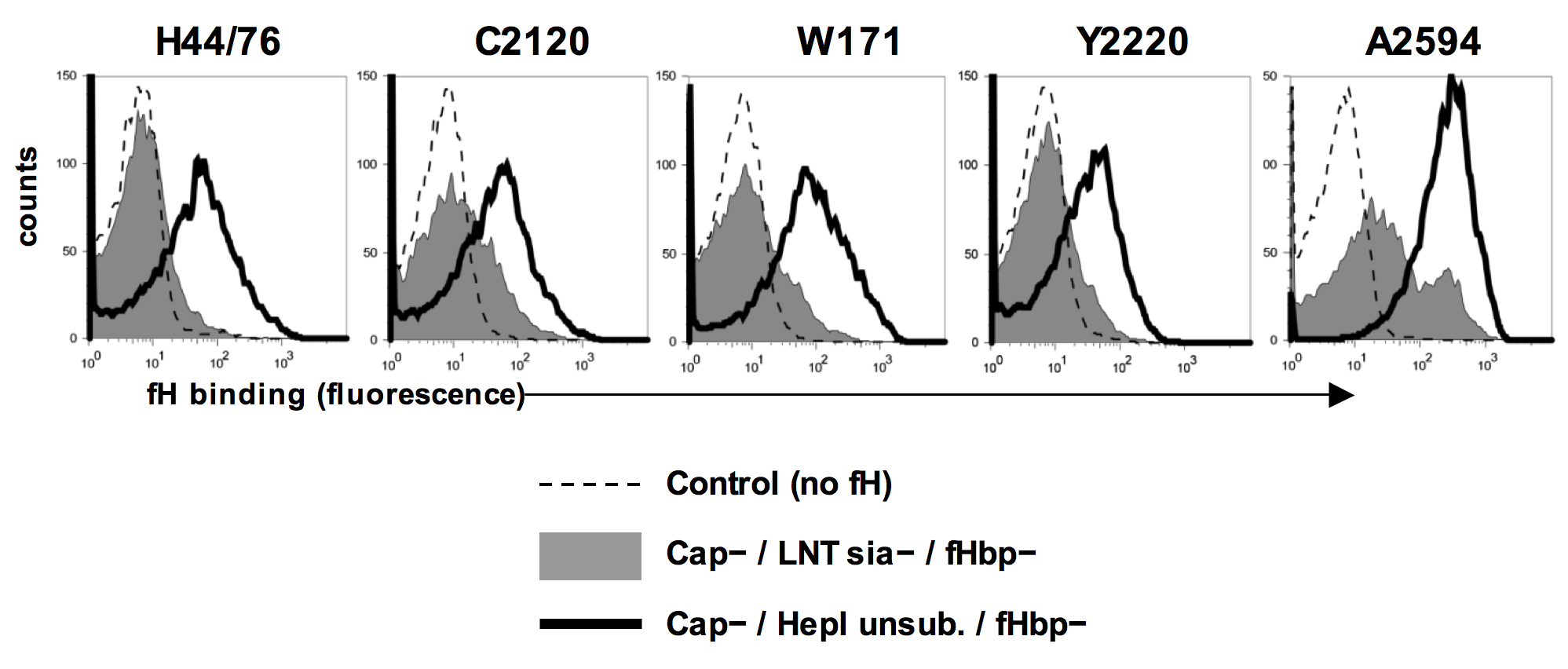

Supplement: Figure S3 — Truncating the HepI chain of LOS in low and intermediate NspA expressing strains discloses fH binding. fH (10 µg/ml) binding to the Cap−/LNT sia− mutants of strains H44/76, C2120, W171 and Y2220 were compared to their isogenic mutants that lacked glycan extensions from HepI (HepI unsubstituted). (0.28 MB TIF) [file ppat.1001027.s003.tif]

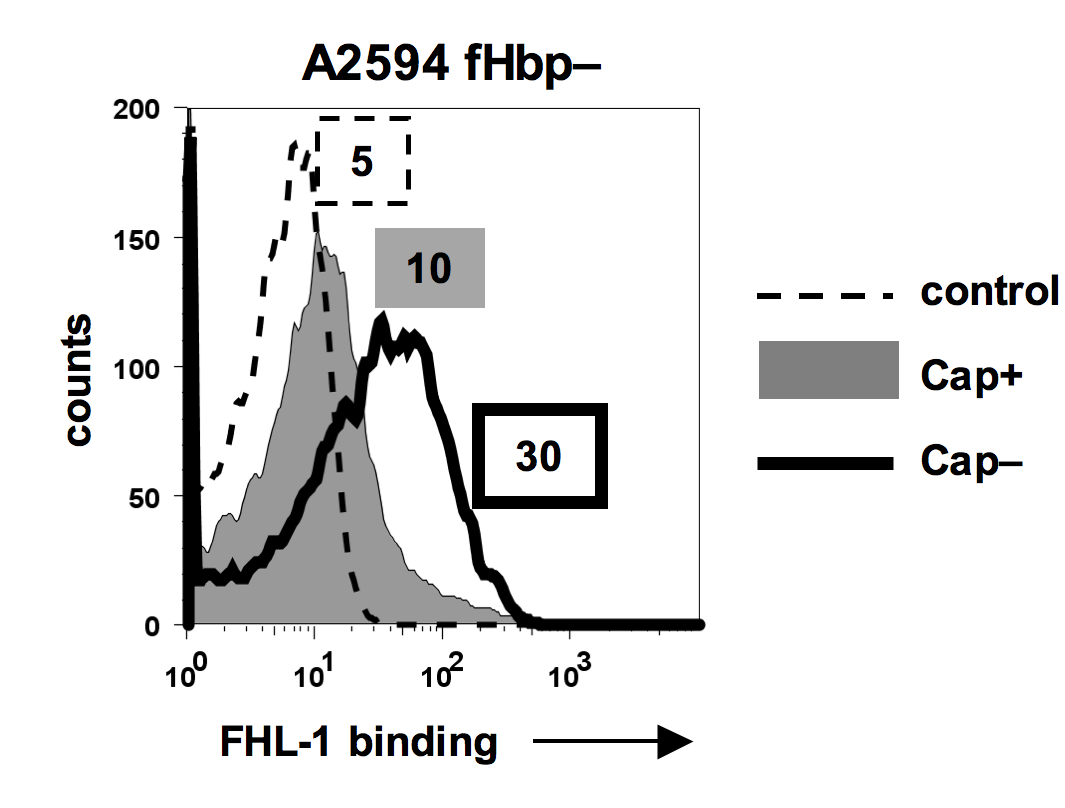

Supplement: Figure S4 — Binding of purified recombinant FHL-1 (7µg/ml) to encapsulated (gray shaded histogram) and unencapsulated (solid black line) N. meningitidis strain A2594 that expresses NspA but not fHbp. In all graphs, the x-axis represents fluorescence on a log10 scale and the y-axis the number of events. Numbers represent the median fluorescence of the corresponding histogram. Purified FHL-1 was omitted from control reaction mixtures (broken line). (0.11 MB TIF) [file ppat.1001027.s004.tif]

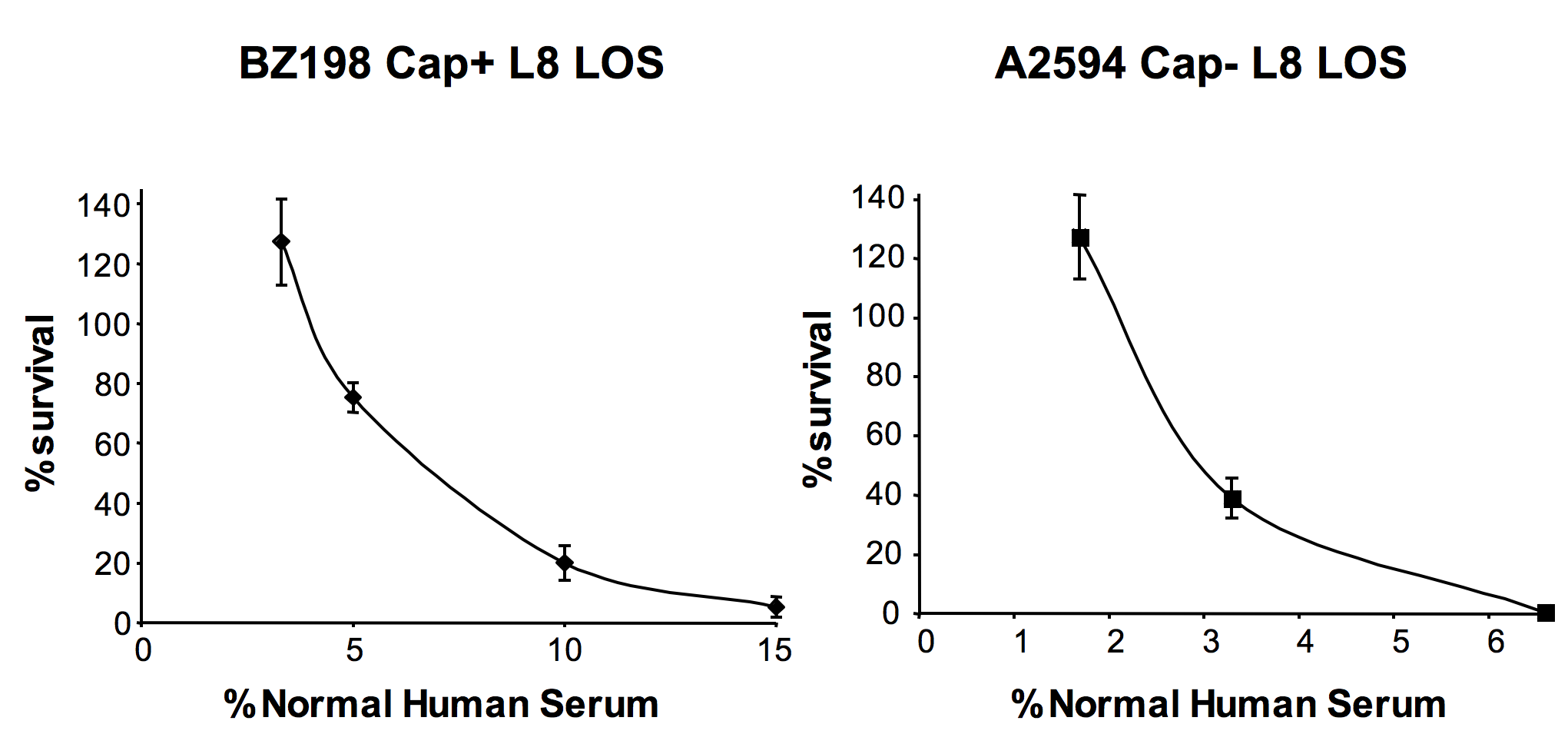

Supplement: Figure S5 — Titration of serum concentrations to determine the level of serum resistance of BZ198 Cap+ L8 LOS and A2594 Cap− L8 LOS. Strains BZ198 Cap+ L8 LOS (left graph) and A2594 Cap− L8 LOS (right graph) were tested for their ability to resist killing by normal human serum in a serum bactericidal assay. The y-axis represents percent survival and the x-axis represents the percent serum used in the assay. Error bars indicate standard deviation calculated from 3 independent experiments. (0.15 MB TIF) [file ppat.1001027.s005.tif]
