# Supplementary material for: The Meningococcal Vaccine Candidate Neisserial Surface Protein A (NspA) Binds to Factor H and Enhances Meningococcal Resistance to Complement
Source: PLoS Pathog. 2010 Jul 29;6(7):e1001027. doi: 10.1371/journal.ppat.1001027 (PMC2912398; doi:10.1371/journal.ppat.1001027)
Supplement: Table S1 — Estimated mean of normalized fH binding and p-values for trend test. (0.05 MB DOC) [file ppat.1001027.s006.doc]

**Supplementary Table S1**. Estimated mean of normalized fH binding and p-values for trend test.

| Strain | LOS | Mean* of the median fluorescence (standard deviation) | Mean of normalized fH binding (95% CI)** | p-value for trend test |
| --- | --- | --- | --- | --- |
| A2594 Cap+ fHbp- | no fH control | 3.67 (1.15) |  |  |
|  | LNT | 5.67 (2.08) | 1.53 (-6.27, 9.34) | 0.007 |
|  | L8 | 58.33 (19.66) | 17.84 (10.04, 25.65) |  |
|  | unsubstituted | 122.00 (7.94) | 17.84 (10.04, 25.65) |  |
|  |  |  |  |  |
| A2594 Cap- fHbp- | no fH control | 4.33 (0.58) |  |  |
|  | LNT | 41.33 (15.04) | 9.88 (5.636, 14.14) | 0.007 |
|  | L8 | 129.00 (3.61) | 30.17 (25.91, 34.42) |  |
|  | unsubstituted | 169.33 (17.62) | 39.18 (34.93, 43.44) |  |
|  |  |  |  |  |

* calculated from three independent experiments.

** normalized to background binding.
