# Supplementary material for: The Meningococcal Vaccine Candidate Neisserial Surface Protein A (NspA) Binds to Factor H and Enhances Meningococcal Resistance to Complement
Source: PLoS Pathog. 2010 Jul 29;6(7):e1001027. doi: 10.1371/journal.ppat.1001027 (PMC2912398; doi:10.1371/journal.ppat.1001027)
Supplement: Table S2 — Meningococcal strains used in this study and their relevant characteristics. (0.15 MB DOC) [file ppat.1001027.s007.doc]

**Supplementary Table S2.**  Meningococcal strains used in this study and their relevant characteristics

| **Strain name** | **Designation** | **Genotype** | **Description** | **Reference** |
| --- | --- | --- | --- | --- |
| A2594 | A2594 Cap+ / LNT sia- |  | A:4:1-9:ST-5, Cap+, LOS: LNT→HepI (sia-) | [12] |
| A2594 fHbp | A2594 Cap+ / LNT sia- / fHbp- | *fHbp::erm* | *fHbp* deletion mutant of A2594; Cap+; LOS: LNT→HepI (sia-) | This study |
| A2594 lgtA fHbp | A2594 Cap+ / L8 LOS / fHbp- | *fHbp::erm lgtA::kan* | *fHbp* deletion mutant of A2594; Cap+; LOS: lactose→HepI (sia-) | This study |
| A2594 lgtF fHbp | A2594 Cap+ / HepI unsub. / fHbp- | *fHbp::erm lgtF::Spc* | *fHbp* deletion mutant of A2594; encapsulated; LOS: HepI unsubstituted | This study |
| A2594 mynB | A2594 Cap- / LNT sia- | *mynB::Cm* | Cap- mutant derivative of A2594; LOS: LNT→HepI (sia-) | [12] |
| A2594 mynB fHbp | A2594 Cap- / LNT sia- / fHbp- | *mynB::Cm fHbp::erm* | *fHbp* deletion mutant of Cap- A2594; LOS: LNT→HepI (sia-) | This study |
| A2594 mynB lgtA | A2594 Cap- / L8 LOS | *mynB::Cm lgtA::kan* | Cap- mutant of A2594; LOS: lactose→HepI | This study |
| A2594 mynB lgtA fHbp | A2594 Cap- / L8 LOS / fHbp- | *mynB::Cm lgtA::kan fHbp::erm* | *fHbp* deletion mutant of Cap- A2594; LOS: lactose→HepI | This study |
| A2594 mynB lgtF fHbp | A2594 Cap- / HepI unsub. / fHbp- | *mynB::Cm lgtF::Spc fHbp::erm* | *fHbp* deletion mutant of Cap- A2594; LOS: HepI unsubstituted | This study |
| A2594 mynB lgtA fHbp NspA | A2594 Cap- / LNT sia- / fHbp- / NspA- | mynB::Cm *Cm lgtA::kan fHbp::erm nspA::spc* | *nspA* and *fHbp* deleted from Cap- A2594; LOS: lactose→HepI | This study |
| A2594 mynB lgtA fHbp NspA / NspA_A2594_ comp | A2594 Cap- / LNT sia- / fHbp- / NspA-/NspA_A2594_ comp | *mynB*::*Cm lgtA::kan fHbp::erm nspA::spc* pFP12NspA*_A2594_* (TetM) | *NspA+ complemented in trans*; *chromosomal nspA* and *fHbp* deleted from Cap- A2594; LOS: lactose→HepI | This study |
| A2594 mynB PorA | A2594 Cap- / LNT sia- / PorA- | mynB::Cm *porA::Kan* | *porA* deleted from A2594 Cap- / LNT sia- | This study |
| A2594 mynB PorB3 | A2594 Cap- / LNT sia- / PorB3- | mynB::Cm *porB3::erm* | *porB3* deleted from A2594 Cap- / LNT sia- | This study |
| BZ198 | BZ198 Cap+ / LNT sia+ |  | B:NT:PNST:ET-154 | [29,56] |
| BZ198 fHbp | BZ198 Cap+ / LNT sia+ / fHbp- | *fHbp::erm* | *fHbp* deletion mutant of BZ198; Cap+; LOS: LNT→HepI (sia+) | This study |
| BZ198 NspA | BZ198 Cap+ / LNT sia+ / NspA- | *nspA::spc* | *nspA* deletion mutant of BZ198; Cap+; LOS: LNT→HepI (sia+) | [56] |
| BZ198 fHbp NspA | BZ198 Cap+ / LNT sia+ / fHbp- / NspA- | *fHbp::erm nspA::spc* | *fHbp* and *nspA* deleted from BZ198; Cap+; LOS: LNT→HepI (sia+) | This study |
| BZ198 lgtA | BZ198 Cap+ / L8 LOS | *lgtA::kan* | Cap+ BZ198; LOS: lactose→HepI | This study |
| BZ198 lgtA fHbp | BZ198 Cap+ / L8 LOS / fHbp- | *lgtA::kan fHbp::erm* | *fHbp* deleted from BZ198; Cap+; LOS: lactose→HepI | This study |
| BZ198 lgtA NspA | BZ198 Cap+ / L8 LOS / NspA- | *lgtA::kan nspA::spc* | *nspA* deleted from BZ198; Cap+; LOS: lactose→HepI | This study |
| BZ198 lgtA fHbp NspA | BZ198 Cap+ / L8 LOS / fHbp- / NspA- | *lgtA::kan fHbp::erm nspA::spc* | *fHbp* and *nspA* deleted from BZ198; Cap+; LOS: lactose→HepI | This study |
| Z2087 | Z2087 Cap+ / LNT sia- |  | Serogroup A; Clone IV-I; LOS: LNT→HepI (sia-) | [57,58,59] |
| Z2087 fHbp | Z2087 Cap+ / LNT sia- / fHbp- | *fHbp::erm* | *fHbp* deletion mutant of Z2087; Cap+; LOS: LNT→HepI (sia-) | This study |
| Z2087 mynB | Z2087 Cap- / LNT sia- | *mynB::Cm* | Cap- mutant derivative of Z2087; LOS: LNT→HepI (sia-) | This study |
| Z2087 mynB fHbp | Z2087 Cap- / LNT sia- / fHbp- | *mynB::Cm fHbp::erm* | *fHbp* deletion mutant of Cap- Z2087; LOS: LNT→HepI (sia-) | This study |
| H44/76 | H44/76 Cap+ / LNT sia+ |  | B:15:P1.7,16:ST-32; Norway, 1976; Cap+; LOS: LNT→HepI (partially sia+) | [60,61] |
| H44/76 fHbp | H44/76 Cap+ / LNT sia+ / fHbp- | *fHbp::erm* | *fHbp* deletion mutant of H44/76; Cap+; LOS: LNT→HepI (partially sia+) | [12] |
| H44/76 siaD lst fHbp | H44/76 Cap- / LNT sia- / fHbp- | *siaD::Cm lst::kan fHbp::erm* | Cap- derivative of H44/76; *fHbp* deleted; LOS: LNT→HepI (sia-) | [12] |
| H44/76 siaD lst lgtF fHbp | H44/76 Cap- / HepI unsub. / fHbp- | *siaD::Cm lst::kan lgtF::Spc fHbp::erm* | Cap- derivative of H44/76; *fHbp* deleted; LOS: HepI unsubstituted | This study |
| C2120 siaD lst fHbp | C2120 Cap- / LNT sia- / fHbp- | *siaD::Cm lst::kan fHbp::erm* | Cap- derivative of C2120 (C:NT:P1.5,2:ST-11); *fHbp* deleted; LOS: LNT→HepI (sia-) | This study |
| C2120 siaD lst lgtF fHbp | C2120 Cap- / HepI unsub. / fHbp- | *siaD::Cm lst::kan lgtF::Spc fHbp::erm* | Cap- derivative of C2120; *fHbp* deleted; LOS: HepI unsubstituted | This study |
| W171 siaD lst fHbp | W171 Cap- / LNT sia- / fHbp- | *siaD::Cm lst::kan fHbp::erm* | Cap- derivative of W171 (W-135:NT:P1.10:ST-11); *fHbp* deleted; LOS: LNT→HepI (sia-) | This study |
| W171 siaD lst lgtF fHbp | C2120 Cap- / HepI unsub. / fHbp- | *siaD::Cm lst::kan lgtF::Spc fHbp::erm* | Cap- derivative of W171; *fHbp* deleted; LOS: HepI unsubstituted | This study |
| Y2220 | Y2220 Cap+ / LNT sia+ |  | Y:21:P1.15:ST-172 | [44] |
| Y2220 siaD lst | Y2220 Cap- / LNT sia- | *siaD::Cm lst::kan* | Y2220 Cap-, LOS: LNT→HepI sia- | [44] |
| Y2220 siaD lst fHbp | Y2220 Cap- / LNT sia- / fHbp- | *siaD::Cm lst::kan fHbp::erm* | Y2220 Cap-, LOS: LNT→HepI sia-, *fHbp* deleted | This study |
| Y2220 siaD lst lgtF fHbp | Y2220 Cap- / HepI unsub. / fHbp- | *siaD::Cm lst::kan fHbp::erm lgtF::Spc* | Y2220 Cap-, LOS: HepI unsubstituted sia-, *fHbp* deleted | This study |
| Y2220 lgtA | Y2220 Cap+ / L8 LOS | *lgtA::kan* | Y2220 Cap+; LOS: lactose→HepI sia | This study |
| Y2220 NspA++ | Y2220 / LNT sia+ / NspA++ | *nspA* driven by *porA* promoter | Y2220; Cap+; LOS: LNT→HepI (sia+); *nspA* driven by *porA* promoter and expressed at high levels | This study |
| Y2220 lgtA NspA++ | Y2220 / L8 LOS / NspA++ | *lgtA::kan*; *nspA* driven by *porA* | Y2220; Cap+; LOS: lactose→HepI; *nspA* driven by *porA* promoter and expressed at high levels | This study |

Cap+, encapsulated; Cap-, unencapsulated

Hep, heptose

LNT, lacto-N-neotetraose

NT, nonserotypeable; NST, nonserosubtypeable

sia+ LNT LOS sialylated

sia- LNT LOS not sialylated
